# Digital transformation in the Shared Socioeconomic Pathways

**DOI:** 10.1038/s44168-025-00283-w

**Published:** 2025-08-22

**Authors:** Yee Van Fan, Charlie Wilson, Marina Andrijevic, Henrik Carlsen, Somya Joshi

**Affiliations:** 1 Environmental Change Institute, University of Oxford, Oxford, UK; 2 International Institute for Applied Systems Analysis (IIASA), Laxenburg, Austria; 3 Stockholm Environment Institute, Stockholm, Sweden

**Keywords:** Scientific community, Social sciences

## Abstract

Digital transformation refers to the widespread use of digital technologies in ways that reshape societal and economic activity, with significant impacts on sustainable development and climate challenges—both for better and for worse. Using statistical models calibrated to historical evidence in 62 countries across 12 world regions, we project future digital transformation within the Shared Socioeconomic Pathways (SSPs), adding contextual richness to this scenario framework used extensively in global climate research. In some scenarios, we find a pervasive and prolonged digital divide with up to 45% of the assessed population by mid-century still residing in countries with relatively low levels of digital transformation despite ever-deepening digitalisation in wealthier countries. We set out six use cases for how our explicit representation of digital transformation within the SSPs enables quantitative assessment of digitalisation’s impact on energy, emissions, climate policy, and Sustainable Development Goals. We also discuss challenges with using empirically calibrated models to project digital transformation given its rapid evolution and socioeconomic implications.

## Introduction

The digital era is changing norms in communication, business and investment, policy reform and social interaction. Digitalisation is the use of data and digital technologies^1^, ranging from the internet, cloud computing, and social media platforms to automation, digital twins, and artificial intelligence. Digitalisation improves process and system efficiency, enables data-driven decision-making, and enhances effective monitoring and reporting of climate-related action^2^. On the flip side, digital technologies may also amplify climate-related and other environmental risks, as well as societal risks. These include the displacement of workers due to automation, the spread of misinformation, heightened cybersecurity vulnerabilities, increased electricity consumption from expanding information and communication technology (ICT) infrastructure and a rapid increase in electronic waste and other environmental footprints (energy, water, critical minerals)^3^.

Digital transformation means the increasing penetration of, and dependence on, digital technologies across economic sectors, government and public services, and societal functions^1^, including information dissemination. The speed and extent of digital transformation varies globally, but with a clear general trajectory towards both widening applications and deepening dependence^4,5^.

Scenario assessments help explore and understand potential futures for digital transformation and its implications for climate change mitigation and adaptation^6^. In turn, this facilitates proactive climate risk mitigation, helping society adapt to new realities while ensuring the benefits of digitalisation are widely accessible. This aligns with the Intergovernmental Panel on Climate Change (IPCC) Working Group III report^7,8^, which emphasises that digitalisation supports decarbonisation only if appropriately governed. The EU’s twin transition agenda^9^ further reinforces the entwined green and digital transitions while taking key actions to prevent tensions between them. Similarly, the German Advisory Council on Global Change (WBGU)^10^ underscores the urgent need for formative political action to ensure that digitalisation serves sustainable development and that a growing digital divide is contained. The importance is also reflected in the Global Digital Compact’s emphasis on aligning digital cooperation with sustainability goals (e.g. addressing digital divides)^11^.

Projecting alternative futures for digital transformation is more complex than in other clearly defined sectors with tangible outputs. As a general-purpose technology with applications across sectors, activities and scales, digitalisation poses unique measurement challenges. There is no single consistent way to quantify physical output, performance or levels of digital transformation. A range of different proxy variables have been proposed in current and historical assessments including: ICT infrastructure (e.g. the number of secure internet servers, installation of industrial robots or robot intensity); access and use (e.g. individuals using the internet, number of devices, broadband and mobile subscriptions); innovation and economic activities (e.g. the number of patents, ICT service exports); knowledge and literacy (e.g. ICT skills); and composite metric-based indicators such as the E-Government Development Index (EGDI) (Supplementary Table 1). Though introduced for other purposes, these variables are also useful for exploring potential future digital transformation within the framework of Shared Socioeconomic Pathways (SSPs).

The SSPs define different reference scenarios for global and regional socioeconomic development based on five narratives that span a range of plausible futures and examine resulting challenges and opportunities for climate mitigation and adaptation. The five SSPs are defined by different assumptions on high-level drivers of change: economic growth (GDP), demographics (population), and urbanisation rates. A wide range of additional elements are characterised in both narrative and quantitative forms by numerous studies^12–14^ that interpret the implication of the five SSP narratives for the energy system, land use change, pollution and health, and greenhouse gas (GHG) emissions (Table 1).

**Table 1:** Summary of basic SSP elements^24^ relevant to digital transformation and proposed extension to include digital transformation explicitly

Storyline element		SSP1	SSP2	SSP3	SSP4	SSP5
Main Dimensions of Uncertainty Varied and Quantified in SSP Framework	Economy growth	High in LIC, MIC; medium in HIC^a^	Medium, uneven	Slow	Low in LIC, Medium in other countries	High
	Population growth	Relatively low	Medium	High; Low in Rich OECD	Relatively High; Low in Rich OECD	Relatively low
	Education	High	Medium	Low	V.low-low; Medium in Rich OECD/uneq.	High
	Urbanisation level	High	Medium	Low	High; Medium in Rich OECD	High
	Trade	Moderate	Moderate	Strongly constrained	Moderate	High
	Technology development	Rapid	Medium, uneven	Slow	Rapid in high tech economies and sectors; Slow in others	Rapid
	Technology transfer	Rapid	Slow	Slow	Little within countries to poorer population	Rapid
	Energy technological change	Directed away from fossil fuels, toward efficiency and renewables	Some investment in renewables but continued reliance on fossil fuels	Slow tech change, directed toward domestic energy sources	Diversified investments including efficiency and low-carbon sources	Directed toward fossil fuels; alternative sources not actively pursued
	Carbon intensity	Low	Medium	High in regions with large domestic fossil fuel resources	Low/Medium	High
	Energy intensity	Low	Uneven, higher in LICs	High	Low/Medium	High
Proposed SSP Extension	Relative digital transformation level	Medium-High	Medium-Low	Low	Medium-Low	High
	Digital inequality (divide) between countries^b^	Medium-Low	Medium-Low	High	High-Medium	Low

SSP1 ‘Sustainability’ and SSP5 ‘Fossil-fuelled Development’ represent relatively optimistic trends for human development focused on green growth and energy-intensive economic growth, respectively. SSP3 ‘Regional Rivalry’ (a fragmented world of resurgent nationalism) and SSP4 ‘Inequality’ are more pessimistic, with SSP2 ‘Middle of the Road’ providing a moderate pathway in which future trends broadly follow their historical pattern.

^a^HIC, MIC, LIC = high-, medium-, low-income countries.

^b^% countries/population in very low or low digital transformation categories.

More recently, several studies have extended the SSP narratives and their constituent elements to assess implications for governance^15^, extreme poverty^16^, gender inequality^17^, income inequality^18^, net migration and remittances^19^, structural change^20^, the human development index^21^ and armed conflict risk^22,23^, which involve highly nonlinear phenomena. Resulting ‘SSP extension variables’ are typically based on statistical relationships observed historically with SSP drivers and elements that can then be used to generate future projections based on how these drivers and elements change under the different SSP storylines. All these SSP extensions are designed to enrich the SSP narratives and strengthen their usefulness without altering their fundamental architecture or quantitative interpretation.

Despite how widespread digitalisation is today and its anticipated role as a transformative force shaping economic and social life, it is not explicitly mentioned in the SSP narratives. (One small exception is a mention of digitalisation in the SSP5 narrative with reference to global institutions and coordination^24^).

This remarkable omission is evident both in the SSP storylines and in their quantitative elements and interpretations, including the thousands of scenarios using the SSP framework that were reviewed in the IPCC’s recent 2022 assessment^25^. As a dimension of technological change, digitalisation is implicitly included in the broader context of technological development that varies across the SSPs (Table 1), but this is concerned primarily with energy-related innovation that has a more direct bearing on GHG emissions.

Digital transformation introduces significant GHG mitigation opportunities and challenges through the economic and social activities it enables, as well as its direct energy consumption footprint. The lack of quantitative elements in the SSP framework makes it difficult to assess the enabling or exacerbating effect of digitalisation in uncertainty analyses of climate mitigation futures, including those used to inform policies for achieving national net-zero commitments under the Paris Agreement.

In this study, we assess global trends in digital transformation and explore alternative plausible futures within the SSP framework. We model the historical relationship between socioeconomic and demographic variables (our independent variables) and digital transformation measured by a widely-used UN index^26^ (our dependent variable), across countries and through time. These statistical models guide our projections of future digital transformation, taking into account variation across the SSP storylines.

As an extension to the SSP framework, our quantitative projections show relative differences in the pace and depth of digital transformation between countries in an uncertain long-term future. Coupled to interpretive narratives informed by an expert workshop, our projections enrich the plausible futures characterised by the SSPs and expand their applicability to assess the implications of digitalisation for energy use, material consumption, GHG emissions, and the achievability of climate targets.

The fast-moving pace and varied application of digital technologies make it hard to anticipate future digital transformation, particularly in the long-term^27^. These uncertainties are magnified by the potential for disruptive technological breakthroughs such as generative AI, or even future artificial general intelligence (AGI)^28^.

Despite these uncertainties, the highly consequential nature of digitalisation for climate change invites initial efforts to fill a knowledge gap that can be critiqued and strengthened within the large SSP community^29^. We contribute our results as an enabling framework, including six defined use cases for further quantitative analysis of GHG mitigation and adaptation challenges affected directly or indirectly by digitalisation under the societal, economic and demographic influences on global development explored by the SSPs. An important stream of further analysis would specifically examine future discontinuities with observed historical trends, building on initial analytical work in this area^30^.

## Results

### Digital transformation historically

Given its extensive scope and complexity, digital transformation and its dimensions can be conceptualised in many ways. We use the UN’s EGDI^26^ as our dependent variable. The EDGI combines information from three subindices measuring: ICT infrastructure, access, and use; digital skills proxied by human capital; and online service availability and accessibility (Supplementary Table 4). Compared to other indices or measures summarised in Supplementary Table 1, the EGDI has better representativeness by covering multiple dimensions of digital transformation, and provides broader temporal and spatial coverage.

**Fig. 1:**
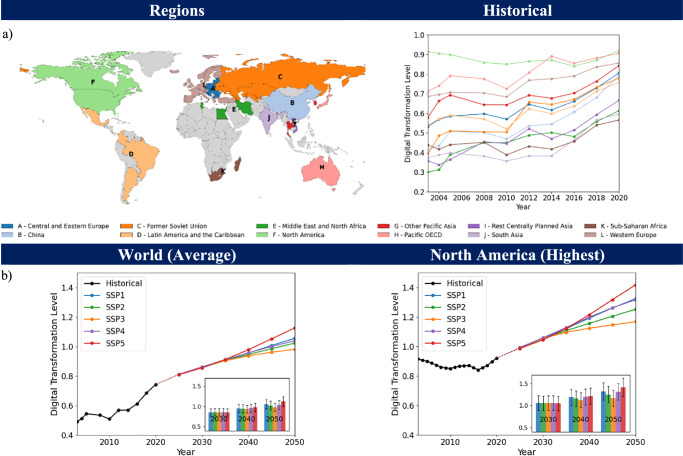
Relative digital transformation level of 12 world regions. **a** Historical digital transformation level based on the E-Government Development Index (EGDI) with representative countries shown for each region, and **b** Projected digital transformation levels up to 2050 for world average (population-weighted) and North America as an example region. Countries shown in grey on the map were not included in the direct analysis. Solid lines show central estimates. The inset highlights digital transformation levels at 3 selected years, along with upper and lower bounds representing 95% confidence intervals for the predicted value based on the uncertainty in estimated coefficients. This shows modest differentiation across SSPs over the long term (see text for discussion).

Using the EGDI as a measure of digital transformation, we show historical trends aggregated to 12 world regions common to global SSP modelling. Our aggregations use population-weighted averages for countries in each region (Fig. 1a). Over the period to 2020, the digital transformation level of North America is generally higher than that of the other 11 regions, with the Pacific OECD catching up and Sub-Saharan Africa the lowest.

We tested a range of socioeconomic and demographic factors as predictors (Supplementary Table 5) of country-level digital transformation using a panel dataset of 62 countries over the period 2003–2020. Our econometric model shows that digital transformation historically can be explained by GDP per capita, R&D expenditure, and population size as independent variables with fixed entity (country) effects to control for unobserved heterogeneity (see ‘Methods’ and Supplementary Table 6), such as trust and cultures.

These independent variables in our model proxy the economic capacity (GDP/capita), technological innovation (R&D) and market demand (population) that drive digital transformation. As well as controlling for the wide variation in country size in our sample, the population variable proxies not just aggregate demand for digital services but also the availability of human capital.

### Future digital transformation within the SSP framework

Using country-level projections for the three independent variables in our model, we project digital transformation levels to 2050 under each of the five SSP narratives. We limit our projections to 2050 as uncertainties amplify over longer time periods, particularly for fast-changing phenomena like digitalisation. However, our model can be used to project out to 2100 if needed to link digital transformation to long-term carbon budgets (see use case discussions and Supplementary Fig. 1).

Our projections of digital transformation go beyond the 0–1 range over which the historical EGDI is normalised, so they should not be interpreted as future values of the EGDI (Supplementary Table 4). However, our measure of digital transformation serves the same function in showing variation between countries and over time (‘Methods’ and Supplementary Table 10). As with the EGDI, absolute values of digital transformation are hard to interpret as they are based on a basket of different subindices and indicators. It is more meaningful to interpret quantitative changes in a relative sense: for example, digital transformation in Sweden is 0.94 in 2020, increasing to 1.25 by 2050, which is in the top percentile of all 62 countries worldwide in our sample. Higher values of digital transformation imply more ICT technologies and infrastructure, stronger human capital and digital skills, and more widespread access to digital services.

Our projections of digital transformation show disparities across storylines as expected (Fig. 1b; Supplementary Fig. 1 and Supplementary Excel Sheet 4 for other regional projections). SSP3 (‘Regional Rivalry’) shows the lowest level and SSP5 (‘Fossil-fuelled Development’) the highest, aligning with its storyline of rapid economic and technological progress. North America demonstrates the highest level of digital transformation, generally above the population-weighted world average, based on 62 countries representing 12 regions with available data. Using North America’s 2020 digital transformation level (0.92) as a benchmark, we can use our projections to estimate when other world regions will reach or surpass that level under different scenario assumptions. In an SSP2 future, for example, regions will ‘catch up’ by 2025 (Pacific OECD, Western Europe), by 2035 (centrally planned Asia, former Soviet Union), or by 2050 or later (Latin America, Middle East). The longest lag is for Sub-Saharan Africa, which surpasses North America’s 2020 digital transformation level only by 2085. It is important to note that for Sub-Saharan Africa, the Middle East and North Africa, and Other Pacific Asia, as presented in Supplementary Fig. 1, the subset of countries included in the analysis represents less than 70% of each region’s total GDP when compared to the 180 countries dataset used for out of sample generalisation projections (Supplementary Information—Supplementary Table 14). Within this subset, Sub-Saharan Africa and Other Pacific Asia are more strongly represented by countries with relatively higher GDP per capita, while the Middle East and North Africa are primarily represented by countries with lower GDP per capita. As such, projections for these regions should be interpreted with some caution due to limitations in regional coverage.

**Fig. 2:**
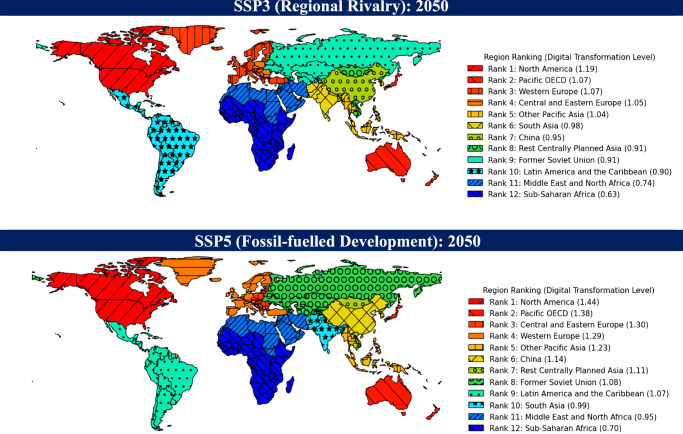
Digital transformation by 2050. Regional ranking under SSP3 (Regional Rivalry) and SSP5 (Fossil-fuelled Development) scenarios based on projections for 62 representative countries. The values in parentheses in the legend represent the digital transformation levels corresponding to their respective ranks. For Sub-Saharan Africa, the Middle East and North Africa, and Other Pacific Asia, the countries included in the analysis account for less than 70% of each region’s total GDP when compared to the full 180 country dataset (see Supplementary Information). In this subset, Sub-Saharan Africa and Other Pacific Asia are predominantly represented by countries with higher GDP/capita, while the Middle East and North Africa are represented by countries with lower GDP/capita.

Ranking the twelve regions by their 2050 digital transformation levels in SSP3 and SSP5 shows both convergence and divergence (Fig. 2). The two highest and the two lowest ranked regions remain unchanged by 2050 (North America and Pacific OECD, and Middle East and Sub-Saharan Africa, respectively). Despite Sub-Saharan Africa being represented by countries with relatively high GDP per capita—potentially leading to an overestimation of its digital transformation ranking—it consistently ranks lowest across scenarios, suggesting that the overall regional ranking remains valid. Some regions, such as South Asia, show a significant change in rank by 2050 (to sixth in SSP3 but tenth in SSP5). This is primarily due to the slower population growth assumed for South Asia under the SSP5 assumptions. These relative changes in digital transformation levels over time between regions can be used in various ways as reference points or modifiers when assessing the implications of digital transformation for GHG mitigation and adaptation challenges (see ‘Discussion’).

### Uneven digital transformation across world regions

**Fig. 3:**
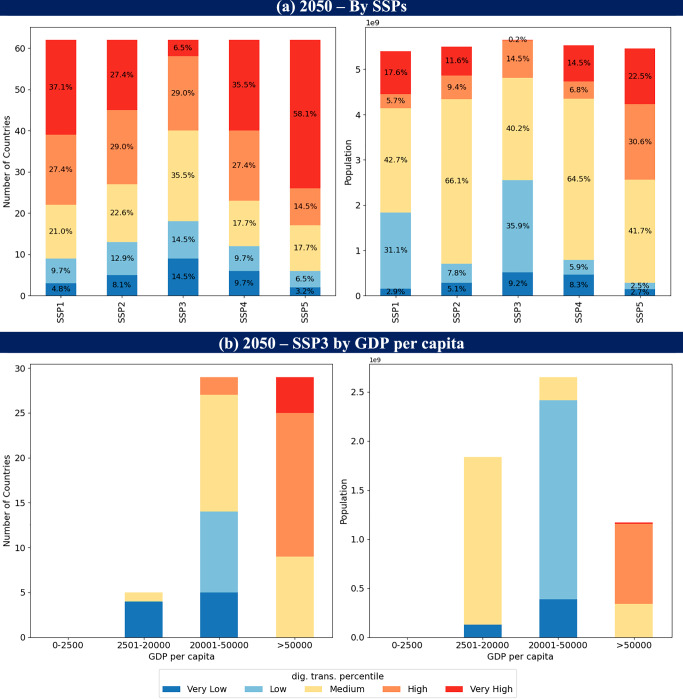
Number of countries and population by digital transformation level in 2050 based on our 62-country sample. **a** SSP1-5 and **b** SSP3 by GDP per capita. The percentile-based categorisation is based on the 2020–2050 digital transformation level: Very high (>90th percentile), high (>75th and ≤90th percentile), medium (>50th and ≤75th percentile), low (>25th and ≤50th percentile), very low (≤25th percentile), see Supplementary Table 11.

We use percentile categories^15^ to group countries into five categories from very low to very high digital transformation level based on the distribution of projected values from 2020 to 2050 across all the assessed countries. A country is classified as relatively ‘very high’ if its digital transformation value exceeds the 90th percentile (>1.22)—meaning its level is in the top 10% of all observed values regardless of scenario (Supplementary Table 11). By mid-century, the proportion of countries categorised as having low (blue) or very low (light blue) levels of digital transformation ranges from 9.7% (under SSP5 ‘Fossil-fuelled Development’) to 29% (under SSP3 ‘Regional Rivalry’) (see Fig. 3a and Supplementary Table 12). This translates into 5–45% of the population of the 62 countries in our sample living with relatively low levels of digital transformation in 2050. Conversely, the share of the population in countries with high (orange) or very high (red) levels of digital transformation ranges from 15 to 53%.

The average differences between SSPs shown in Fig. 3 can strongly exacerbate underlying inequalities if these are a salient feature of the SSP storylines—as with SSP3. Figure 3b shows that among the 45% of the assessed population with low or very low levels of digital transformation under SSP3 assumptions, none are in countries with average GDP per capita over $50,000, and the majority are in countries with GDP per capita between $20,000–$50,000. These trends in the digital divide between high-income and low-income countries are generally consistent across different SSPs, although varying in magnitude.

These results for our 62-country sample (Fig. 3) are biased towards countries with data availability. To provide a full projected global picture, we extend our dataset to 180 countries, but with caution in interpreting these out-of-sample projections (see Supplementary Tables 13 and 14, Supplementary Fig. 8, for approach and limitations). By mid-century, under SSP3 assumptions, we find that 47% of all countries and 35% of the world’s population (~3.5 billion) in 180 countries (see Supplementary Fig. 2a) have access to very low or low levels of digital transformation compared to the other countries.

### Digital transformation as an extension to the SSP narratives and elements

The SSPs comprise both qualitative narratives (or storylines) and quantitative drivers, elements, and other variables. Our quantitative projections of digital transformation in each of the SSPs are summarised in Table 1 alongside the most relevant SSP elements, including demographics, economic development and technological change^24^.

For example, SSP3 ‘Regional Rivalry’ is characterised by relatively weak digital transformation and high digital inequality—a digital divide—in which a significant proportion of the population (~45%) remains at low or very low levels of digital transformation. We also show how digital inequality interacts with income inequality across SSPs with different economic growth and convergence assumptions. For example, SSP3 has the highest proportion of the population facing digital inequality even in countries with higher GDP per capita (using USD 20,000 as a threshold). This is consistent with a storyline in which economic growth does not automatically translate into digital progress, with digital stagnation exacerbating socioeconomic inequalities.

Drawing on insights from an expert workshop on digital and climate futures (see ‘Methods’), we also provide qualitative interpretations of these projections that emphasise how digital transformation interacts with other aspects of the SSP narratives (Supplementary Table 2). As an example, in the SSP4 (‘Inequality’) narrative, digitalisation amplifies the high-growth global knowledge economy with rapid structural change among ‘winning’ countries, firm types, and population segments, but with strong negative effects on job losses, skills displacement, and income polarisation as well as the concentration of power undermining political agency.

## Discussion

Digitalisation is not currently represented within the SSP framework. Our quantitative projections of digital transformation levels across countries and time allow the impacts of digitalisation on GHG mitigation and adaptation challenges to be modelled and assessed consistently and comparatively across SSPs. This adds an important new dimension to the characterisation of reference or baseline scenario uncertainty in global development relevant to climate futures. Explicit representation of digital transformation also improves the SSP framework’s wider policy relevance beyond climate, in line with recommendations by O’Neill^6^. Digital transformation is an integral element of Sustainable Development Goals (SDGs)^31^ on innovation, labour markets, and accessible infrastructure (SDG8,9), and a cross-cutting enabler of many other goals, including those on education and cities (SDG4,11).

Here we set out five use cases for how our projections can enrich understanding of GHG mitigation pathways and policies, with an illustrative sixth use case for GHG adaptation (Table 2).

**Table 2:** Applications of projected digital transformation levels within the Shared Socioeconomic Pathways (SSPs)

Use case	Quantitative elements needed for assessment
1. Assessing direct energy and material consumption and resulting GHG emissions from ICT infrastructure.	Digital Transformation within SSPs [our projections].
	Energy and material intensity or incremental expansion of ICT infrastructure.
	SSP variables include C intensity of electricity and the global distribution of manufacturing and infrastructure.
2. Assessing the indirect impact of specific digital applications (e.g. teleworking) on energy consumption and resulting GHG emissions through changes in behaviour, economic activity, or societal functions.	Digital Transformation within SSPs [our projections].
	Indirect impact assessment of net energy-reducing (substitution, efficiency, optimisation) and net energy-increasing (rebound, induced demand) effects of specific digital applications.
	SSP variables on technological change, economic growth, plus specific variables related to the use of digital applications (e.g. for teleworking: share of services in economic output, urbanisation, travel distances).
3. Assessing the indirect impact (enabling effect) of digitalisation as a general-purpose technology on energy consumption and resulting GHG emissions.	Digital Transformation within SSPs [our projections].
	Statistical relationships between digitalisation and economy-wide (or industry sector) energy demand, controlling for country characteristics.
	SSP variables on country characteristics (e.g. GDP, trade intensity, economic structure).
4. Assessing the interaction between digital transformation and the stringency of climate policy required to reach emission reduction targets.	Digital Transformation within SSPs [our projections].
	Levels of carbon price or other measures of climate policy for reaching defined climate stabilisation targets (e.g. 2 °C) or emission targets (e.g. net-zero).
	SSP variables on country characteristics affecting policy capacity (e.g. government effectiveness) and digitalisation (e.g. R&D intensity).
5. Informing global initiatives to ensure universal and equitable access to digital transformation opportunities as part of the SDG agenda.	Digital Transformation within SSPs [our projections].
	Linkages between digitalisation and SDG outcomes.
	Metrics of digital divide by socioeconomic group, marginal cost of expanding access to ICT infrastructure.
6. Assessing digital climate services for adaptation planning.	Digital Transformation within SSPs [our projections].
	Measures of climate impact and adaptation planning or resilience strategies (e.g. drought frequency and intensity, climate impact on agricultural yields, agricultural diversification, improved access to markets).
	Links between digital climate services and adaptation planning (e.g. weather and climate prediction tools, yield modelling tools, geospatial analysis of climate vulnerabilities, ICT availability to farmers).

### Assessing direct energy and material consumption and resulting GHG emissions from ICT infrastructure

The data centres, networks and devices that comprise ICT infrastructure have a direct energy consumption footprint estimated at 1000 TWh in 2023, equivalent to 4% of global electricity use^32^. This is projected to increase rapidly in the next 5 years in some locations^33^ due to energy-intensive training and inference of generative AI, including large language models. The material needs of ICT infrastructure are small relative to bulk material flows globally, but account for significant shares of certain critical minerals, including rare earths like indium, gallium and germanium^34^. Our long-term projections of digital transformation levels can be coupled to analyses of the energy and/or material consumption of the continuing expansion of ICT infrastructure across countries and regions under SSP storylines. For example, the marginal effect of digital transformation on energy demand growth observed historically (controlling for other drivers of demand) could be estimated across different time periods and regions. Resulting elasticities of ICT energy demand to deepening digital transformation in turn would enable longer-term projections under different scenario assumptions, including those in the SSPs. Existing SSP variables refine this analysis by contextualising the carbon intensity of electricity and manufacturing activities across different regions (Table 2). Insights on future energy consumption and GHG emissions from ICT infrastructure across countries under SSP storylines, as well as the extent of dependence on rare earth materials, water or recoverable electronic waste, inform policymakers concerned with resource uncertainties and resilience linked with digital trends.

### Assessing the indirect impact of specific digital applications (e.g. teleworking) on energy consumption and resulting GHG emissions through changes in behaviour, economic activity or societal functions

Digitalisation impacts energy consumption and GHGs indirectly by influencing or changing household and firm behaviour and so the structure of social and economic activity. Compared to direct impacts, indirect impacts are larger in magnitude, more uncertain, and harder to model for methodological reasons, including difficulties in clearly defining system boundaries^35,36^. Impacts can be net energy-reducing (through substitution, efficiency, optimisation) or net energy-increasing (through rebound, induced demand)^37^. Our projections of the relative levels of digital transformation across different regions provide a means of scaling and extrapolating estimates of indirect impacts from specific digital applications. For example, Hook et al.^38^ synthesised data showing that teleworking results in a net reduction in overall energy use in transport and buildings sectors ranging from −15% to −0.01% accounting for variation in study design, context, and geography. These estimates of indirect impacts of energy can be combined with SSP variables capturing heterogeneity in relevant adoption conditions across countries (Table 2) to project future indirect impacts of specific digital applications on energy, consistent with SSP storylines. This helps understand their contribution to mitigation goals or the mitigation challenges they pose.

### Assessing the indirect impact (enabling effect) of digitalisation as a general-purpose technology on energy consumption and resulting GHG emissions

Econometric models identify relationships between digital transformation and energy demand across the whole economy or in industrial sectors. For example, Briglauer et al.^39^ found a small but significant negative elasticity of CO_2_ emissions with respect to broadband connections in OECD countries over the period 2002–2019. Kopp et al.^40^ found that a 10% increase in firms’ ICT investments was associated with a 0.29% decrease in emissions, with a stronger effect in higher-income countries. These statistical models of the aggregate energy impacts of digitalisation typically control for variation in country size, development stage, trade relationships, and other factors explaining energy demand or GHG emissions. A general finding is that additional digitalisation reduces energy demand at the margins, with a stronger effect in more developed economies. These elasticities identified historically can be integrated with our projected levels of digital transformation across different regions to understand the future economy-wide impact of digitalisation for mitigation goals within different SSPs (Table 2).

### Assessing the interaction between digital transformation and the stringency of climate policy required to reach emission reduction targets

By design, SSP reference scenarios assume no additional or new climate policies, and vary widely in GHG emission trends resulting from SSP drivers and other elements. This allows climate policy assumptions to be layered in to identify the stringency required to achieve net-zero or other defined emission reduction or climate stabilisation targets under different SSP uncertainties^25^. As digitalisation will have significant direct and indirect impacts on energy demand, varying across sector and region, digital transformation will interact with climate policy assessments as both enabler and potential exacerbator. Our projected digital transformation levels allow this to be explored explicitly and quantitatively, including by specifying the relationship between the extent of digitalisation and the carbon pricing levels (or the de-risking of finance) needed to achieve net-zero targets. SSP variables related to governance conditions and effectiveness^15^ provide additional context, helping to tailor policy insights to the specific capacities of different regions^41^ (Table 2).

### Informing global initiatives to ensure universal and equitable access to digital transformation opportunities as part of the SDG agenda

Digital transformation plays a fundamental but complex role in progressing towards SDGs^31,42^. Global modelling analyses quantify the synergies and trade-offs of different strategies across the SDGs^43^ but have not been able to include the interacting effects of digitalisation. Our projected digital transformation levels allow analysis of relationships between SDG indicators and digitalisation across countries under SSP storyline uncertainties. An important example is the digital divide between countries and over time that is shown clearly in our projections (Fig. 3). Modelling the progression of this divide, and how it can be strategically tackled through digital access and infrastructure investments can improve understanding policy capacity (and coherence) to address specific SDG concerns associated with digitalisation (Table 2).

### Assessing digital climate services for adaptation planning

Effective climate change adaptation depends not only on traditional resilience strategies but also on the use of digital technologies to improve responsiveness and decision-making. Our example use cases for SSP-consistent projections of digital transformation levels have emphasised impacts on GHG mitigation challenges and opportunities (via energy demand and GHG emissions). However, there are many examples of digital applications both strengthening adaptation planning to climate impacts^44^ (e.g. early warning systems for extreme weather events) but also adversely affecting resilience (e.g. over-dependence on digital infrastructure, digital divides). Our projections can be used to explore how digitalisation influences climate change, adaptive capacity and adaptation planning. For example, empirical studies that show how farmers’ practices are affected by access to accurate weather prediction models or real-time information on market prices for agricultural commodities can be coupled to our projections of how the underlying digital capacities to access and use such tools vary across countries and time (Table 2). This adds a new dimension to SSP-consistent analysis of climate adaptation challenges while emphasising how marked regional variation in digital transformation interacts with the geography of climate impacts. The digital divide potentially adds an additional vulnerability to impact hot spots already subjected to multiple climate stressors^45^.

The digital transformation level projects change in future digital infrastructure, activities, and services, as well as the capacity, including human capital, to support increasing digitalisation. While high digital transformation has the potential to enable sustainable development and net-zero transition pathways, it does not guarantee such outcomes. Digitalisation has its own energy and resource consumption footprint, exacerbated by high carbon intensities of electricity in some futures (e.g. SSP5). Digitalisation also has powerful adverse as well as beneficial impacts across application domains and economic sectors, from governance and societal interactions to industrial processes, jobs and livelihoods.

There are two important limitations and considerations for our SSP-consistent projections of digital transformation. The first concerns uncertainty; the second concerns endogeneity.

Projections based on econometric models using panel data assume that historical relationships observed between variables will hold in the future. While these models can often account for dynamic changes in the short term, uncertainties compound and amplify over the long term. This is particularly the case for digital transformation given its fast-moving innovation cycles, speed of deployment, and potential for surprises^27^. Our main projections only run to 2050 and emphasise uncertainty around our central estimates.

Some of these uncertainties relevant to digitalisation are represented in the SSP framework and the quantitative projections of drivers, elements and extension variables in the different SSPs. These include rates of technological change and economic growth, and between-country inequalities (Table 1) that indirectly flow through into our projections via their impact on the independent variables in our model.

However, additional uncertainties are specific to digitalisation, particularly those related to breakthroughs in AI^46^ that may result in discontinuous change or even systemic disruption^30^, rather than the smooth path-dependent trajectories projected by our historically calibrated panel models using future GDP, population, and R&D intensity trends.

Against these smooth trajectories, the effects of AI, generative AI, or other digital advances not captured in historical relationships can be explored using sensitivity analysis, sector-specific projections, or further what-if scenario analysis—all of which can be nested within the SSP framework’s long-term variation of macro uncertainties relating to the economy, demography, urbanisation, and technological change.

These issues of uncertainty are arguably less fundamental than issues of endogeneity. Our approach treats digital transformation as an internally consistent outcome of socioeconomic development already characterised by different SSPs. However, it is not only an outcome. Digital transformation is also a driver or amplifier of socioeconomic uncertainty, as our analysis of the digital divide compounding income inequality shows. At the same time, digitalisation can contribute to productivity gains and accelerate economic growth.

This two-way endogenous relationship—digitalisation as both driver and outcome of change—was also emphasised in our expert workshop mapping of linkages between digital transformation and SSP elements (Supplementary Table 3).

As our approach takes the SSP framework as given—both as a set of future narratives and as derived demographic and economic variables used in our modelling—we do not capture the feedback effects of digital transformation on social and economic development. The same limitation applies (by design) to climate impacts on the socioeconomic development pathways, which determine GHG emissions but do not co-evolve with the resulting changes in climate.

Other SSP extensions, such as gender inequality and the rule of law, also face this endogeneity problem as they both impact and are impacted by socioeconomic development. As with our projections of digital transformation, the value of these SSP extensions is in making important uncertainties explicit in order to open up further avenues for policy-relevant analysis (see our use cases). What is distinctive about digital transformation, however, is the speed and magnitude of its potential disruptiveness.

As an example, digital transformation as a driver of change may undermine governance institutions and political agency through misinformation, unchecked market power of tech companies, and social polarisation under assumptions of weak global oversight^3^. Generative AI could turbocharge this challenge to the governance landscape necessary for concerted action on global commons problems like climate change, highlighting risks similar to the tragedy of the commons^47^. To some extent, this is captured implicitly in the SSP3 storyline of fragmentation, divergence, and weakened global institutions. But could AI accelerate and amplify this effect to the point at which it destabilises the fundamental socioeconomic assumptions and relationships on which the SSP framework is built? This eventuality is explored by Carlsen et al.^30^ in the case of a breakthrough on AGI^28^. Resulting systemic disruption could require a reimagining of a wholly new scenario architecture for understanding future uncertainties relevant to climate goals.

## Methods

Our methodology (Supplementary Fig. 3) begins with the pre-selection of independent variables hypothesised to have a relationship with our digital transformation dependent variable. We then build stepwise regression models testing the predictive ability of each independent variable (Supplementary Table 5), yielding a viable historical model for SSP-consistent future projections. Our emphasis in the specification of this historical model is to identify independent variables for which SSP-consistent projections either already exist (e.g. GDP growth as a core SSP driver) or can be estimated indirectly through their observed associations with SSP drivers or elements (e.g. R&D intensity as a function of GDP growth). We can then couple our viable historical model to SSP-consistent projections of the independent variables to estimate within-country change over time in digital transformation in each of the five SSP pathways. Finally, we propose simple narratives to help interpret these quantitative projections of digital transformation, drawing on stakeholder input collected from an expert workshop.

### Historical data

Our dependent variable is the EGDI^26^, which we use as an indicator of digital transformation across diverse countries. Published by the UN, the EDGI has the advantage of broad representativeness (extending beyond ICT infrastructure) and the availability of panel data, both in terms of year and country coverage. It combines three components—the telecommunication infrastructure index, the human capital index and the online service index—into a weighted average of normalised scores (Supplementary Table 4). The EGDI is not designed to capture development in an absolute sense; rather, it aims to provide a comparative performance rating of countries. This is consistent with the purpose of our study. The EGDI consists of components that assess the readiness of the digital economy, including infrastructure, human capital, and the prevalence and acceptance of online services. For model estimation, we use data for 62 countries for which there are no missing observations for any variables over the timeframe 2003–2020.

To present historical data (and projected results), we group countries into 12 world regions, matching those commonly used by the global Integrated Assessment Models used in quantitative analyses of SSPs (Supplementary Fig. 4). We use population-weighted averages to aggregate digital transformation from countries to world regions. Some regions are represented by a small number of countries—for example, Sub-Saharan Africa is represented by two countries that tend to have a higher GDP per capita than the regional average. This is further detailed in Supplementary Fig. 4 and Supplementary Information. To expand country coverage, we conducted out-of-sample generalisation based on 180 countries instead of the original 62. However, since the model was trained on only 62 countries, we present these extended 180 country results only as Supplementary Information, with potential limitations of this generalisation discussed and visualised.

Our independent variables are a range of country-level economic, technological and sociodemographic characteristics associated with digital transformation, drawing primarily on data from the World Bank (Supplementary Table 5).

### Historical model

We use stepwise panel data regression to add or remove independent variables systematically based on criteria including statistical significance (*p* < 0.05) and hypothesised associations with the dependent variable (Supplementary Table 5). Multicollinearity between independent variables was a persistent problem, particularly with GDP per capita and other economic measures. We use a variance inflation factor (VIF) threshold of <5, iterating between alternative model specifications to find a good model fit with clear interpretability. We test and compare ordinary least squares, fixed effects (fixed entity or fixed time), and random effect models. We use the Breusch-Pagan Lagrange Multiplier test and the Hausman test to statistically justify the appropriateness of each model, supported by theoretical considerations (Supplementary Table 6).

We select a fixed effect model as our preferred final viable model with the form shown in Eq. (1). The overall model has an *R*^2^ = 0.777 (F-statistic = 86.279, *p* < 0.01), with coefficients given in Table 3. The independent variables are GDP per capita, population, and R&D intensity (Supplementary Table 6).

1 $$Digital\,Transformatio{n}_{it}={\alpha }_{i}+{\beta }_{1}GD{P}_{it}+{\beta }_{{2}}Po{p}_{it}+{\beta }_{3}R\& {D}_{it}+{\varepsilon }_{it}$$

in which ${\alpha }_{i}$ represents the fixed effect for country $i$, ${\varepsilon }_{{it}}$ is the error term for country $i$ at time *t*, ${\beta }_{1}$ = coefficient for GDP per capita, PPP (2017), ${\beta }_{2}$ = coefficient for population, ${\beta }_{3}$ = coefficient for R&D expenditure (% GDP).

**Table 3:** Fixed effect model—digital transformation (EGDI) historically

Independent variables	VIF (multicollinearity)	Coefficient	*p* value
GDP per capita, PPP (2017)	3.807 (<5)	1.04e-05	0.0000
Population	1.099 (<5)	0.0472	0.0001
R&D expenditure (% of GDP)	3.997 (<5)	8.296e-10	0.0003

We validated our model fitted to 2003–2020 observations using the 2022 dataset as a test year to compare actual versus projected values. Overall, the projection error remained below 3%, and regional rankings were unchanged or in two cases differed by one rank. Details are provided in Supplementary Fig. 7 and Supplementary Table 7. We conduct additional global sensitivity analysis on the explanatory variables using permutation importance and moment-independent importance measure (see Supplementary Table 8). This shows GDP per capita to be the most influential variable on model projections of digital transformation. This is consistent with expectations given the role of economic growth in driving both supply of digital infrastructure and demand for digital services, as well as enabling human capital (the three constitutive elements of the EGDI).

### SSP-consistent projections

In our future projections we refer to ‘digital transformation levels’ as our dependent variable to distinguish it from the historical EGDI which has a scale of 0–1. Our future projections are not constrained by this cap (Supplementary Table 10).

We project future changes in relative levels of digital transformation in line with the five SSPs using projected future values of our independent variables extracted from the SSP Public Database or its extension explorer^48^. However, not all independent variables have projected future SSP data available, e.g. R&D expenditure (Eq. (1) and Supplementary Table 5). In such cases, we follow the approach used by Leimbach et al.^20^ which uses historical regression to explain non-SSP variables as a function of variability in core SSP elements, particularly GDP. The steps and models involved in this procedure, including robustness checks, are shown in Supplementary Fig. 3 (Step 4) with results and robustness checks in Supplementary Fig. 5 and Supplementary Table 9.

Our SSP-consistent projections of digital transformation levels are limited by their reliance on historical relationships with predictors (GDP per capita, population, R&D expenditure) and fixed entity effects. They do not capture uncertainties not evident in the historical observations such as the impact of crises or disruptive innovations (e.g. AI growth).

Our projections run from 2020 to 2050 at 5-year intervals with indicative extended projections to 2100 in Supplementary Fig. 1. Within the SSP framework and its quantification, most of the SSP elements are modelled by using historical data from 1980 to 2015, with projections extending from 2020. We use 2020 as a calibration point to align observations with the projected data. This alignment updates the SSP data for consistency with recent trends, an approach also utilised in Hoy et al.^49^. Full details of the alignment procedure are in Supplementary Fig. 6.

We categorise digital transformation levels into percentiles-based group using the projected distribution of digital transformation across all scenarios from 2020 to 2050, with the cut-off for each category stated in Supplementary Table 11. The five categories are defined as: very high (>90th percentile), high (>75th and ≤90th percentile), medium (>50th and ≤75th percentile), low (>25th and ≤50th percentile), and very low (≤25th percentile). A country with a score or level above 90th percentile is classified as ‘very high’, meaning its digital transformation level is higher than 90% of all country-year scores across 2020–2050 period. Countries in the ‘very low’ category fall within the lowest 25%. This categorisation is used to analyse the distribution of countries and their populations under different scenarios, along with their associated GDP per capita.

### SSP narratives

O’Neill et al.^24^ is the principal reference point for SSP narrative descriptions. Since the SSP narratives nor derived quantifications capture digitalisation trajectories, we interpret our projections of relative digital transformation level alongside the storylines described in O’Neill et al.^24^ and extend these storylines specifically for digitalisation (Supplementary Table 2). For this, we draw on insights from stakeholders at an expert workshop on digitalisation and climate narratives (Supplementary Table 3). The workshop^50^ was held on May 13–14, 2024 and brought together 35 researchers and practitioners working on digitalisation’s impacts on energy, the economy, markets, society, and governance.

## Supplementary information

R2 Supplementary Materials_R1 CW_E_Clean

Supplementary Excel_Clean

## Data Availability

Data are provided within the Supplementary Information files. The Python code (standard regression analysis) used in this study is available from the corresponding author upon request.

## References

[1] Vectors of digital transformation. OECD, https://www.oecd.org/en/publications/vectors-of-digital-transformation_5ade2bba-en.html (2019).

[2] Digitalization and energy—analysis. IEA, https://www.iea.org/reports/digitalisation-and-energy (2017).

[3] Creutzig, F. et al. Digitalization and the Anthropocene. *Annu. Rev. Environ. Resour.* **47**, 479–509 (2022).

[4] Measuring digital development: facts and figures 2024. ITU, https://www.itu.int:443/en/ITU-D/Statistics/pages/facts/default.aspx.

[5] *Digital Progress and Trends Report: Interactive Charts—Digital Adoption* World Bank, https://www.worldbank.org/en/data/interactive/2024/03/04/digital-progress-and-trends-report-interactive-charts.

[6] O’Neill, B. C. et al. Achievements and needs for the climate change scenario framework. *Nat. Clim. Change* **10**, 1074–1084 (2020). 10.1038/s41558-020-00952-0 PMC7688299 33262808

[7] Intergovernmental Panel on Climate Change. *Climate Change 2022 Mitigation of Climate Change* https://www.ipcc.ch/report/ar6/wg3/chapter/summary-for-policymakers/ (2022).

[8] Pathak, M. et al. *Climate Change 2022 Mitigation of Climate Change: Technical Summary* (Cambridge University Press, 2022). 10.1017/9781009157926.002.

[9] The twin green & digital transition: How sustainable digital technologies could enable a carbon-neutral EU by 2050—European Commission. https://joint-research-centre.ec.europa.eu/jrc-news-and-updates/twin-green-digital-transition-how-sustainable-digital-technologies-could-enable-carbon-neutral-eu-2022-06-29_en.

[10] *Towards Our Common Digital Future* https://www.wbgu.de/en/publications/publication/unsere-gemeinsame-digitale-zukunft.

[11] Homepage | Global Digital Compact. https://www.un.org/global-digital-compact/en.

[12] Riahi, K. et al. The Shared Socioeconomic Pathways and their energy, land use, and greenhouse gas emissions implications: an overview. *Glob. Environ. Change* **42**, 153–168 (2017).

[13] Bauer, N. et al. Shared socio-economic pathways of the energy sector—quantifying the narratives. *Glob. Environ. Change* **42**, 316–330 (2017).

[14] Rao, S. et al. Future air pollution in the shared socio-economic pathways. *Glob. Environ. Change* **42**, 346–358 (2017).

[15] Andrijevic, M., Crespo Cuaresma, J., Muttarak, R. & Schleussner, C.-F. Governance in socioeconomic pathways and its role for future adaptive capacity. *Nat. Sustain.* **3**, 35–41 (2020).

[16] Crespo Cuaresma, J. et al. Will the Sustainable Development Goals be fulfilled? Assessing present and future global poverty. *Palgrave Commun.* **4**, 1–8 (2018).

[17] Andrijevic, M., Crespo Cuaresma, J., Lissner, T., Thomas, A. & Schleussner, C.-F. Overcoming gender inequality for climate resilient development. *Nat. Commun.* **11**, 6261 (2020). 33319776 10.1038/s41467-020-19856-w PMC7738534

[18] Rao, N. D., Sauer, P., Gidden, M. & Riahi, K. Income inequality projections for the Shared Socioeconomic Pathways (SSPs). *Futures* **105**, 27–39 (2019).

[19] Benveniste, H., Cuaresma, J. C., Gidden, M. & Muttarak, R. Tracing international migration in projections of income and inequality across the Shared Socioeconomic Pathways. *Clim. Change* **166**, 39 (2021).

[20] Leimbach, M., Marcolino, M. & Koch, J. Structural change scenarios within the SSP framework. *Futures* **150**, 103156 (2023).

[21] Cuaresma, J. C. & Lutz, W. The demography of human development and climate change vulnerability: a projection exercise. *Vienna Yearb. Popul. Res.* **2015**, 241–262 (2016).

[22] Hoch, J. M. et al. Projecting armed conflict risk in Africa towards 2050 along the SSP-RCP scenarios: a machine learning approach. *Environ. Res. Lett.* **16**, 124068 (2021).

[23] Hegre, H. et al. Forecasting civil conflict along the Shared Socioeconomic Pathways. *Environ. Res. Lett.* **11**, 054002 (2016).

[24] O’Neill, B. C. et al. The roads ahead: Narratives for Shared Socioeconomic Pathways describing world futures in the 21st century. *Glob. Environ. Change* **42**, 169–180 (2017).

[25] Riahi, K. et al. Mitigation pathways compatible with long-term goals. In *Climate Change 2022 - Mitigation of Climate Change Working Group III Contribution to the Sixth Assessment Report of the Intergovernmental Panel on Climate Change* (eds Shukla, A. R. et al.) Ch. 3, 295–408 (Cambridge University Press, 2022).

[26] United Nations. E-Government Development Index. https://publicadministration.un.org/egovkb/en-us/About/Overview/-E-Government-Development-Index.

[27] Koomey, J. & Masanet, E. Does not compute: avoiding pitfalls assessing the Internet’s energy and carbon impacts. *Joule* **5**, 1625–1628 (2021).

[28] Suleyman, M. & Bhaskar, M. *The Coming Wave: AI, Power and the Twenty-First Century’s Greatest Dilemma* (Bodley Head, 2023).

[29] Gritsenko, D., Aaen, J. & Flyvbjerg, B. Rethinking digitalization and climate: don’t predict, mitigate. *npj Clim. Action* **3**, 1–7 (2024).

[30] Carlsen, H., Nykvist, B., Joshi, S. & Heintz, F. Chasing artificial intelligence in Shared Socioeconomic Pathways. *One Earth* **7**, 18–22 (2024).

[31] Vinuesa, R. et al. The role of artificial intelligence in achieving the Sustainable Development Goals. *Nat. Commun.* **11**, 233 (2020). 31932590 10.1038/s41467-019-14108-y PMC6957485

[32] Energy and AI—analysis. IEA, https://www.iea.org/reports/energy-and-ai (2025).

[33] What the data centre and AI boom could mean for the energy sector—analysis. IEA, https://www.iea.org/commentaries/what-the-data-centre-and-ai-boom-could-mean-for-the-energy-sector (2024).

[34] Malmodin, J., Bergmark, P. & Matinfar, S. A high-level estimate of the material footprints of the ICT and the E&M sector. in 168–148. 10.29007/q5fw.

[35] Bieser, J. C. T., Hintemann, R., Hilty, L. M. & Beucker, S. A review of assessments of the greenhouse gas footprint and abatement potential of information and communication technology. *Environ. Impact Assess. Rev.* **99**, 107033 (2023).

[36] Wilson, C. et al. Evidence synthesis of indirect impacts of digitalisation on energy and emissions. In *Proc. 2024 10th International Conference on ICT for Sustainability (ICT4S)* 116–127. 10.1109/ICT4S64576.2024.00021 (2024).

[37] Pettifor, H., Agnew, M., Wilson, C. & Niamir, L. Disentangling the carbon emissions impact of digital consumer innovations. *J. Clean. Prod.* **485**, 144412 (2024).

[38] Hook, A., Court, V., Sovacool, B. K. & Sorrell, S. A systematic review of the energy and climate impacts of teleworking. *Environ. Res. Lett.* **15**, 093003 (2020).

[39] Briglauer, W., Köppl-Turyna, M., Schwarzbauer, W. & Bittó, V. Evaluating the effects of ICT core elements on CO2 emissions: recent evidence from OECD countries. *Telecommun. Policy* **47**, 102581 (2023).

[40] Kopp, T., Nabernegg, M. & Lange, S. The net climate effect of digitalization, differentiating between firms and households. *Energy Econ.* **126**, 106941 (2023).

[41] Yang, C., Gu, M. & Albitar, K. Government in the digital age: exploring the impact of digital transformation on governmental efficiency. *Technol. Forecast. Soc. Change* **208**, 123722 (2024).

[42] *The Digital Revolution and Sustainable Development: Opportunities and Challenges. Report Prepared by the World in 2050 Initiative* https://pure.iiasa.ac.at/id/eprint/15913/, 10.22022/TNT/05-2019.15913 (2019).

[43] van Soest, H. L. et al. Analysing interactions among Sustainable Development Goals with Integrated Assessment Models. *Glob. Transit.* **1**, 210–225 (2019).

[44] Balogun, A.-L. et al. Assessing the potentials of digitalization as a tool for climate change adaptation and sustainable development in urban centres. *Sustain. Cities Soc.* **53**, 101888 (2020).

[45] Byers, E. et al. Global exposure and vulnerability to multi-sector development and climate change hotspots. *Environ. Res. Lett.* **13**, 055012 (2018).

[46] Luers, A. et al. Will AI accelerate or delay the race to net-zero emissions?. *Nature* **628**, 718–720 (2024). 38649764 10.1038/d41586-024-01137-x

[47] Ostrom, E. Tragedy of the commons. in *The New Palgrave Dictionary of Economics* 13778–13782. 10.1057/978-1-349-95189-5_2047 (Palgrave Macmillan, 2018).

[48] SSP Extensions Explorer. https://ssp-extensions.apps.ece.iiasa.ac.at/.

[49] Hoy, Z. X., Woon, K. S., Chin, W. C., Van Fan, Y. & Yoo, S. J. Curbing global solid waste emissions toward net-zero warming futures. *Science* **382**, 797–800 (2023). 37972189 10.1126/science.adg3177

[50] Expert workshop on digitalization narratives and climate change mitigation. IIASA—International Institute for Applied Systems Analysis. https://iiasa.ac.at/blog/jun-2024/expert-workshop-on-digitalization-narratives-and-climate-change-mitigation.

