# Supplementary material for: Digital transformation in the Shared Socioeconomic Pathways
Source: NPJ Clim Action. 2025 Aug 22;4(1):79. doi: 10.1038/s44168-025-00283-w (PMC12370532; doi:10.1038/s44168-025-00283-w)
Supplement: Supplementary file 1 — R2 Supplementary Materials_R1 CW_E_Clean [file 44168_2025_283_MOESM1_ESM.docx]

*Supplementary Materials*

Digital Transformation in the Shared Socioeconomic Pathways

| Content |
| --- |
| **Supplementary Tables** |
| **Supplementary Table 1:** Single indicators and indices used to measure digital transformation.  **Supplementary Table 2:** Summary of the five SSP narratives with indicative links to digitalisation as a driver, characteristic, or outcome of change.  **Supplementary Table 3:** Links between digitalisation and SSP narrative elements based on an expert workshop.  **Supplementary Table 4:** The E-Government Development Index (EGDI) as a measure of relative digital transformation levels between countries and within countries over time.  **Supplementary Table 5:** Tested independent variables, data sources, and hypothesised relationships with digital transformation (dependent variable).  **Supplementary Table 6:** Comparison of alternative historical model specifications and fits to the data.  **Supplementary Table 7:** Actual and projected regional rankings in 2022. **Supplementary Table 8:** Permutation feature and differential importance.  **Supplementary Table 9:** Historical model explaining R&D expenditure used to project future R&D and relative digital transformation levels.  **Supplementary Table 10:** Interpreting projected relative digital transformation levels.  **Supplementary Table 11:** Categorisation of digital transformation levels based on 2020-2050 data from 62 countries  **Supplementary Table 12:** Digital transformation level percentiles by number of countries and population (62 countries dataset) |
| **Supplementary Figures** |
| **Supplementary Figure 1:** Digital transformation levels in 12 world regions (population weighted) within five shared socioeconomic pathways (SSPs) up to 2100.  **Supplementary Figure 2:** Digital transformation levels (2050) categorised by percentiles across 5 SSPs and by GDP/cap based on 180 countries dataset.  **Supplementary Figure 3:** Workflow for projecting digital transformation levels under SSP-consistent assumptions.  **Supplementary Figure 4:** Assessed countries and world regions.  **Supplementary Figure 5:** Projected future R&D expenditure in 12 world regions (GDP-weighted country aggregations) within five shared socioeconomic pathways (SSPs).  **Supplementary Figure 6:** Aligning historical trends and future projections using 2020 as a calibration point.  **Supplementary Figure 7:** Comparison of the actual and projected digital transformation level in 2022 |
| **Supplementary Information: Out-of-Sample Projections** |
| **Supplementary Table 13:** Countries and socioeconomic indicators included in the extended datasets, with 2020 data used as historical data for model development as an example  **Supplementary Table 14:** The percentage share of the number of countries, GDP, and population of the 62-country dataset compared to the 180-country dataset, categorised by region.  **Supplementary Figure 8:** Cumulative Distribution Function (CDF) plots of GDP per capita and population for the 62-country dataset (red) and the 180-country dataset (blue) in 2020 |
| **Supplementary Excel: Digital Transformation within Shared Socioeconomic Pathways** |
| **Sheet 1:** Relative digital transformation levels for 62 countries and their respective regions (2013-2100, across 5 SSPs).  **Sheet 2:** Fixed effects for 62 countries with corresponding plotted graph.  **Sheet 3:** Relative digital transformation levels for an additional 118 countries (2025-2100, across 5 SSPs).  **Sheet 4:** Relative digital transformation levels at region levels assessed based on two different models in estimating the future R&D expenditure (one of the independent variables) |

**Supplementary Table 1: Single indicators and indices used to measure digital transformation.**

| Indicators of Digital Transformation | | | | |
| --- | --- | --- | --- | --- |
| Single Measures | Available Year^1^ | World Data^1^ | EU Data^1^ | Reference |
| Proportion (%) of total business sector workforce involved in the ICT sector | 2002-2021 | N | Y | UNCTADSTAT |
| Value added in the ICT sector as a percentage of total business sector value added | 2002-2021 | N | Y | UNCTADSTAT |
| International trade in ICT services, value, shares and growth | 2005-2022 | Y | Y | UNCTADSTAT |
| Percentage of the ICT sector in Gross value added (ICT services, ICT manufacturing) | 2012-2121 | N | Y | Eurostat |
| ICT service exports (% of service exports, Balance of Payments) | 2002-2022 | Y | Y | IMF, Worldbank |
| High-technology exports (current US$) | 2007-2022 | Y | Y | Comtrade, Worldbank |
| ICT skills (by type, gender, age) | 2013-2022 | N | Y | ITU |
| Mobile-broadband Internet traffic (within the country) | 2014-2022 | Y | Y | ITU |
| Number of Internet Service Providers (ISP) | 2014-2023 | Y | Y | ITU |
| Secure Internet servers (per 1 million people) | 2010-2021 | Y | Y | Worldbank |
| Number of devices (% of population) | 2014-2022 | Y | Y | ITU |
| ICT goods imports (% total goods imports) | 2000-2021 | Y | Y | Worldbank |
| Fixed broadband subscriptions | 2004-2021 | Y | Y | Worldbank, ITU |
| Mobile cellular subscriptions | 2004-2021 | Y | Y | Worldbank, ITU |
| Individuals using the Internet (% of population) | 1995-2021 | Y | Y | Worldbank, ITU |
| Robot density | 2013-2022 | Y | Y | The Robot Report |
| Digital Skills (Share of ICT specialists in employment) | 2013-2023 | N | Y | Eurostat |
| ICT Access and Usage by Businesses | 2005-2023 | OECD | Y | OECD Data explorer |
| ICT Access and Usage by Households | 2005-2023 | OECD | Y | OECD Data explorer |
| ICT Access and Usage by Individuals | 2005-2023 | OECD | Y | OECD Data explorer |
| ICT Usage by Internet Users | 2005-2023 | OECD | Y | OECD Data explorer |
| ICT prices | 2008-2023 | Y | Y | ITU |
| Indices | | | | |
| E-Government Development Index (comprising: Telecommunication Infrastructure index, Human Capital index, Online Service Index – see Supplementary Table 4) | 2003-2024 | Y | Y | UN E-Government Knowledgebase |
| Digital Economy and Society Index (DESI) | 2014-2022 | N | Y | Eurostat |
| Digital Services Trade Restrictiveness Index | 2014-2022 | OECD | Y | OECD Going Digital Toolkit |
| Digital Government Index | 2023 | OECD | Y | OECD Going Digital Toolkit |
| ICT Development Index | 2009-2017 (discontinued); Restarted in 2023 with revised methodology | Y | Y | ITU |
| Digital Adoption Index | 2014 and 2016 | Y | Y | Worldbank |
| Network(ed) Readiness Index | 2023-2024 | Y | Y | Portulans Institute and University of Oxford |
| AI preparedness index (Digital infrastructure, innovation and economic integration, human capital and labour market policies, regulation and ethics) | 2023 | Y | Y | IMF |
| Frontier technology readiness index (use, adopt, adapt) | 2008-2021 | Y | Y | UNCTADSTAT |
| Mobile Connectivity Index | 2014-2023 | Y | Y | GSMA |
| ^1^Even though 'Yes-Y' and specific years are indicated, this does not imply that the entire dataset (covering all years or all countries globally) is available. Some measures have significant gaps in year or country coverage. | | | | |
| Of the single measures, the number of broadband and mobile subscriptions are the most simplified and widely applied.  Metric-based indicators or indices provide an overall performance of digital development across a wider range of composite dimensions. These include the Digital Adoption Index, with data available for two years, the ICT Development Index, and the Digital Economy and Society Index established by the EU.  Globally, the E-Government Development Index provides insight into relative digitalisation levels within and between countries. The E-Government Development Index is a composite index comprising three components: the Telecommunication Infrastructure Index, the Human Capital Index and the Online Service Index. This latter index measures digital penetration in government and public services, consisting of five sub-indices quantified using survey assessments of relevant institutional frameworks, service provision, content provision, technology and e-participation. As a result, the Online Service Index – one of the indices aggregated into the E-Government Development Index – indirectly measures a country’s digital culture and propensity more widely than indices based on access and infrastructure. | | | | |
| Additional information   - There are other indexes, as summarised by Charfeddine and Umlai (2023), that were developed and published in academic articles without being released as official reports or included in a database. - ITU is one of the primary sources for digitalisation-related indicators, collected and grouped under six major themes: sustainability, connectivity, affordability, governance, markets, and trust. These indicators are compiled from reports at the country level, including those provided by ministries. - This is not an exhaustive list of digitalisation related indicators, but a summary of commonly used measures—both single and indices—that capture digital disparities across different places. There are other single technical indicators that measure the performance of digital technology, such as FlOPS for GPU or CPU, clock speed, internet speed, or RAM latency. They are not included in this table, as such measures are more time-dependent, reflecting technological progress rather than significant regional or country variation. These indicators are specific to digital performance and do not necessarily capture broader aspects of digital transformation, such as infrastructure readiness, digital skills, usage and access, or the socioeconomic factors that drive digital development, , which differs from the purpose of this study. | | | | |
| References   - Charfeddine, L., Umlai, M. (2023). ICT sector, digitization and environmental sustainability: A systematic review of the literature from 2000 to 2022. Renewable and Sustainable Energy Reviews, 184, 113482. - Eurostat, https://ec.europa.eu/eurostat/databrowser - GSMA, https://www.mobileconnectivityindex.com/index.html#year=2023&dataSet=indexScore - International Monetary Fund (IMF), https://www.imf.org/external/datamapper/datasets - International Telecommunication Union (ITU), https://datahub.itu.int/query/ - OECD Data Explorer, https://www.oecd.org/en/data/datasets/oecd-DE.html - OECD Going Digital Toolkit, https://goingdigital.oecd.org/ - Portulans Institute and University of Oxford, https://networkreadinessindex.org/ - The Robot Report, https://www.therobotreport.com - UNCTADSTAT, https://unctadstat.unctad.org/datacentre/ - United Nations Comtrade Database, https://comtradeplus.un.org - World Bank, https://data.worldbank.org/indicator | | | | |

**Supplementary Table 2: Summary of the five SSP narratives with indicative links to digitalisation as a driver, characteristic, or outcome of change.**

| **SSP** | **SSP description: text copied from O’Neill, Kriegler et al. (2016)** | **Indicative links with digital transformation** |
| --- | --- | --- |
| **SSP1: Sustainability—Taking the green road** | "Commitment to achieving development goals, increasing environmental awareness in societies around the world, and a gradual move toward less resource-intensive lifestyles, constitutes a break with recent history in which emerging economies have followed the resource-intensive development model of industrialized countries." | Management of the global commons to respect planetary boundaries extends to the digital sphere, aligning digitalisation with climate governance including through cooperative global institutions and an emphasis on de-materialisation and less resource intensive lifestyles. |
| **SSP2: Middle of the road** | "A development pathway consistent with typical patterns of historical experience observed over the past century. For example, emerging economies grow relatively quickly and then slow as incomes reach higher levels. This growth, along with income inequality that persists or improves only slowly, continuing societal stratification, and limited social cohesion, constrains significant advances in sustainable development." | Digitalisation's historical role as an amplifier and accelerator of economic and social change continues with both positive aspects (e.g., productivity gains) and negative aspects (e.g., inequality of access, digital divide) remaining in tension. |
| **SSP3: Regional rivalry—A rocky road** | "Concerns about competitiveness and security push countries to increasingly focus on domestic issues. This trend is reinforced by comparatively weak global institutions. Countries focus on achieving energy and food security goals within their own regions at the expense of broader-based development. Several regions move toward more authoritarian forms of government with highly regulated economies. Investments in education and technological development decline." | Digitalisation is more tightly focused on national champions and policy goals with a gradual de-globalisation of the digital economy and slower overall rates of digital transformation. |
| **SSP4: Inequality—A road divided** | "Highly unequal investments in human capital, combined with increasing disparities in economic opportunity and political power, lead to increasing inequalities and stratification both across and within countries. Over time, a gap widens between an internationally-connected society that is well educated and contributes to knowledge- and capital-intensive sectors of the global economy, and a fragmented collection of lower-income, poorly educated societies that work in a labour intensive, low- tech economy." | Digitalisation amplifies the high-growth global knowledge economy with rapid structural change among 'winning' countries and population segments, but with strong negative effects on job losses, skills displacement, and income polarisation as well as the concentration of power undermining political agency. [*1] |
| **SSP5: Fossil-fuelled development—Taking the highway** | "Global markets are increasingly integrated, with strong investments in health, education, and institutions to enhance human and social capital. The push for economic and social development is coupled with the exploitation of abundant fossil fuel resources and the adoption of resource and energy intensive lifestyles around the world. All these factors lead to rapid growth of the global economy." | Digitalisation enables accelerated globalisation and rapid development of emerging economies through new opportunities in the knowledge economy, and increasing global integration and convergence of platforms, social media networks, and consumption patterns. [*2] |

*1: The SSP4 narrative alludes to but does not mention digitalisation: "rising inequality is assumed to arise from a number of factors including skill-biased technology development (where technology replaces many low-skill jobs."

*2: The SSP5 narrative is the only one that makes explicit reference to digitalisation: " the digital revolution enables a global discourse of a significant and increasing fraction of the global population for the first time in human history which may lead to a rapid rise in global institutions and promote the ability for global coordination".

**Supplementary Table 3: Links between digitalisation and SSP narrative elements based on an expert workshop.** Summary of insights from an expert workshop of links between digitalisation impacts in four domains (columns) and SSP elements (rows). The four domains are society & behaviour, economy & firms, governance & markets, energy & materials. SSP elements can be linked to digitalisation impacts both as an effect or outcome, and as a cause or driver. The expert workshop on digital futures and climate change was held on May 13-14, 2024, at the International Institute for Applied Systems Analysis (IIASA) in Austria, with 35 participants (scientists and industry).

| **SSP element** | SSP elements can be *the result of*, be caused by, be enabled by digitalisation impacts  or SSP elements can *lead to*, cause, or result in digitalisation impacts. | | | | General link between SSP element and digitalisation impacts, plus *alignment with SSP1,3,4,5 storylines** |
| --- | --- | --- | --- | --- | --- |
| **Domain ⇒**  **SSP element ⇓** | **society & behaviour** | **economy & firms** | **governance & markets** | **energy & materials** |  |
| **Education** | *leads to*: weaker effect of misinformation on polarisation undermining social trust  *leads to*: stronger digital skills and accessibility, reducing digital divide | *leads to*: more digitalisation in knowledge economy |  |  | Education and skills as an enabler of digital skills and knowledge economy and to overcome the digital divide.  *aligns with:* SSP5, and in inverse form with SSP4 |
| **GDP (Economic growth)** |  | *is the result of*: productivity improvements & new job/skills opportunities (supported by retraining) … otherwise is constrained by inequalities | *is the result of*: innovation activity in both directed (towards sustainability) and undirected markets | *is the result of*: cost & time savings, and lower transaction costs (more convenience) | Generally positive effect of digitalisation on economic growth via productivity and innovation.  *aligns with*: SSP1, SSP5 |
| **International trade** |  | *is the result of*: data-driven business models, interoperability & supply chain connectivity across geographies | *leads to*: more global distribution of digital infrastructure |  | Digitalisation both enables and is enabled by economic globalisation.  *aligns with:* SSP1, SSP5 |
| **Regional convergence** | *is the result of*: digital platforms spreading information & influence | *is the result of*: digitally-enabled trade, and digital services  *is the result of*: market concentration in global tech firms | *leads to*: more global distribution of digital infrastructure |  | Digitalisation reduces regional variation via both infrastructure and information flows but with risks of power concentrating in lead firms or markets.  *aligns with:* SSP1, SSP4, SSP5 |
| **Technological change** |  |  | *leads to*: both directed digital innovation towards sustainability and undirected innovation |  | Digitalisation is part of technology development, transfer, and change processes.  *aligns with:* SSP1, SSP5 |
| **Income inequality** |  | *is the result of*: job losses, skills polarisation & market concentration | *leads to*: undermining of social trust and so government effectiveness |  | Exacerbating effect of digitalisation on income and gender inequality via labour markets, with knock-on risks for social cohesion.  *aligns with*: SSP3, SSP4 |
| **Gender inequality** | [see under education & digital divide] | *is the result of*: job losses, skills polarisation | *leads to*: undermining of social trust and so government effectiveness |  |  |
| **Global institutions** |  | *are the result of*: digital governance needs and capabilities  *lead to*: stronger interoperability and standardisation of digital protocols & practices | *lead to*: stronger & more digital governance for directing innovation activity |  | Digitalisation both requires and enables effective global institutions.  *aligns with:* SSP1, SSP5, and in inverse form with SSP3 |
| **Government effectiveness** | *leads to*: strengthened disclosure & transparency of supply chain data (inc. on carbon emissions)  *is the result of*: social trust … otherwise is constrained by polarisation |  | *leads to*: stronger & more digital governance for directing innovation activity  *is the result of*: social trust … otherwise is constrained by inequalities |  | Digitalisation can either enable government effectiveness and rule of law (e.g., via enhanced transparency, individual data rights & sovereignty) or undermine them (e.g., via surveillance, digital divide, and private capture of public goods).  *aligns with:* SSP1 (enabling effect), SSP4 (undermining effect) |
| **Rule of law** |  |  | *leads to*: stronger & more digital governance for directing innovation activity |  |  |
| **Demography** | not included in digitalisation impact pathways | | | | |
| **Structural change** | not included in digitalisation impact pathways | | | | |
| **Urbanisation** | not included in digitalisation impact pathways | | | | |

**Supplementary Table 4: The E-Government Development Index (EGDI) as a measure of relative digital transformation levels between countries and within countries over time.**

| **E-Government Development Index (EGDI)** | **Telecommunication Infrastructure Index** | **Human Capital Index** | **Online Service Index^a^** |
| --- | --- | --- | --- |
| A weighted average of three normalised scores on the Telecommunication Infrastructure Index, Human Capital Index, Online Service Index. Prior to the normalisation of the three component indicators, the Z-score standardisation procedure is implemented for each indicator to ensure their balanced contribution to the overall EGDI. | A composite of five indicators: (i) estimated internet users per 100 inhabitants; (ii) number of main fixed telephone lines per 100 inhabitants*; (iii) number of mobile subscribers per 100 inhabitants; (iv) number of wireless broadband subscriptions per 100 inhabitants; and (v) number of fixed broadband subscriptions per 100 inhabitants. | A composite of four indicators: (i) adult literacy rate; (ii) the combined primary, secondary and tertiary gross enrolment ratio; (iii) expected years of schooling; and (iv) average years of schooling. | A composite of subindices quantified using survey assessments of information and services provided by local governments through official websites. The specific questions used in the assessment of national portals are not disclosed. Quantifications are normalised to a range of 0-1 by subtracting the lowest score and dividing by the difference between the maximum and minimum scores. The latest edition is calculated based on five weighted subindices: (i) institutional framework; (ii) services provision; (iii) content provision; (iv) technology; (v) e-participation. |
| EGDI and its component indicators may show year-to-year volatility due to changes in normalisation anchors and updates to indicator definitions. Our analysis (along with the proposed use cases and cautions against absolute interpretation) like the UN’s own latest reporting (e.g., UN E-Government Survey 2024), interprets relative changes over time using linearised trends. | | | |
| Reference:  United Nations (2024), E-government Development Index: https://publicadministration.un.org/egovkb/en-us/About/Overview/-E-Government-Development-Index  United Nations (2024), E-government Survey 2022: https://publicadministration.un.org/egovkb/en-us  UN E-Government Survey 2020: https://publicadministration.desa.un.org/publications/un-e-government-survey-2020  UN E-Government Survey 2024: https://publicadministration.desa.un.org/publications/un-e-government-survey-2024-0 | | | |

*In some countries, the saturation and decline of fixed-line telephone subscriptions may have been offset or mitigated by a rise in mobile subscriptions. In others, however, the decline in fixed-line access has led to a notable decrease in the Telecommunications Infrastructure Index. Updates to this index are detailed in the UN E-Government Survey 2020.

**Supplementary Table 5: Tested independent variables, data sources, and hypothesised relationships with digital transformation (dependent variable).**

| **Variables** | **Data Source** | **Hypothesised Relationship with Digital Transformation** | **Note** | **SSP** |
| --- | --- | --- | --- | --- |
| Population | World Bank^1^ | Larger populations signify greater market potential, driving investment in and demand for digitalisation. It also relates to the availability of human capital for further supporting digital transformation | Tested*** | Driver^4^ |
| Urbanisation | World Bank^1^ | Higher urbanisation rates facilitate access to and adoption of digital technologies and services | Tested | Driver^4^ |
| GDP per capita | World Bank^1^ | Higher GDP per capita levels indicate greater economic resources available for investment in digitalisation | Tested*** | Driver^4^ |
| Enrolment in tertiary education | World Bank^1^ | Increased tertiary education enrolment enhances workforce skills in digital technology utilisation and innovation. | Tested | Element^4^ |
| Services, Value added | World Bank^1^ | Higher value-added in the service sector indicates a more digitally integrated economy | Tested | Extension^5^ |
| Manufacturing, Value added | World Bank^1^ | Higher value-added in the manufacturing sector indicates a more digitally integrated economy | Tested | Extension^5^ |
| Rule of law | World Bank^2^ | Strong rule of law provides a stable environment conducive to digital innovation and investment | Tested | Extension^5^ |
| Government effectiveness | World Bank^2^ | Effective governance facilitates policies and investments in digital infrastructure and services | Tested | Extension^5^ |
| Renewable electricity output | World Bank^1^ | Higher adoption of renewable energy supports sustainable and cost-effective digital infrastructure | Tested | Energy^6^ |
| Trade openness | World Bank^1^ | Openness to international trade facilitates access to global digital markets and technologies | Tested | No |
| Foreign direct investment | World Bank^1^ | Increased foreign direct investment brings expertise and resources for digital infrastructure development | Tested | No |
| R&D expenditure | World Bank^1^ | Greater investment in research and development drives innovation in digital technologies | Tested*** | No |
| Electricity price (Industry/Household) | IEA^3^ | Competitive electricity prices lower operational costs for digital infrastructure and services | Tested | No |
| High-technology exports | World Bank^1^ | Exporting high-tech products indicates technological advancement and innovation capacity | Tested | No |
| ***=were found to be statistically significant and free from multicollinearity issues (verified via VIF test), and sufficient data is available. | | | | |
| **References & Links** | | | | |
| 1. World Bank, Indicators: https://data.worldbank.org/indicator?tab=all 2. World Bank, Worldwide Governance Indicators: https://www.worldbank.org/en/publication/worldwide-governance-indicators 3. IEA: https://www.iea.org/data-and-statistics/data-tools/end-use-prices-data-explorer?tab=Overview 4. Basic SSP element: https://tntcat.iiasa.ac.at/SspDb/dsd (Latest version: https://data.ece.iiasa.ac.at/ssp/) 5. SSP extension: https://ssp-extensions.apps.ece.iiasa.ac.at/ 6. SSP energy (IAM scenario): https://tntcat.iiasa.ac.at/SspDb/dsd and https://doi.org/10.1016/j.gloenvcha.2016.07.006 | | | | |

**Supplementary Table 6: Comparison of alternative historical model specifications and fits to the data.**

|  | **OLS** | **Fixed Effect** | **Random Effect** | **Mixed Effect** |
| --- | --- | --- | --- | --- |
| Model assumptions | (1) There is no unobserved heterogeneity | (1) Each country has unique attributes that do not change over time and that could influence the dependent variable.  (2) Fixed effects are correlated with the independent variables. | (1) Country-specific effects are random.  (2) Random effects are uncorrelated with the independent variables. | (1) There is both fixed and random variability in the data. |
| Our assessment | (1) Does not hold. | (1) Possible. Examples include institutional quality, policy changes, and cultural attitudes (country fixed effects).  (2) High possibility. Examples of time fixed effects include global technology trends and economic recessions. Unobserved heterogeneity may be correlated with the independent variables. | (1) Possible. Examples of random effects are country-specific shocks (e.g., economic crises, natural disasters).  (2) Possible. No correlation between random effects and independent variables. | (1) Possible (if random effects are shown). |
| Breusch-Pagan test of heteroskedasticity | <0.05, reject the null hypothesis of homoscedasticity. Strong evidence of heteroscedasticity | NA | NA | Not Tested |
| F-statistic | 344 | 86.279 | 200.57 | NA |
| F-test for poolability | NA | *p*-value = 0.0000, reject the null hypothesis of no individual effects (country) | NA | NA |
| Log-Likelihood | 543.60 | 758.72 | 719.24 | NA |
| Hausman test | NA | *p*-value = 0.00057, reject the null hypothesis. Strong evidence that individual effects are correlated with the regressors. The fixed (entity/country) effects model is preferred over the random effects model.  Hausman Test Statistic: 17.463  A significant difference between the estimates from the fixed and random effects models. | | NA |
| R^2^ | 0.626 | 0.777 | 0.834 | Not Tested |
| **Overall Selection** | | | | |
| We select the fixed effects model based on our assessment of the data structure, supported by statistical tests, particularly the Hausman test (p<.01) that shows individual effects are correlated with the regressors. Random effects models generally require larger sample sizes to provide reliable estimates. Moreover, the results are less straightforward and more challenging to clearly interpret. Our assessment and the Breusch-Pagan test suggest the OLS model is not suitable. | | | | |

**Supplementary Table 7: Model validation: actual versus projected regional rankings in 2022**

| **Region** | **Actual Rank** | **Projected Rank** | **Rank Difference** |
| --- | --- | --- | --- |
| North America | 1 | 1 | 0 |
| Pacific OECD | 2 | 2 | 0 |
| Western Europe | 3 | 3 | 0 |
| Other Pacific Asia | 4 | 4 | 0 |
| China | 5 | 6 | 1 |
| Central and Eastern Europe | 6 | 5 | -1 |
| Former Soviet Union | 7 | 7 | 0 |
| Latin America and the Caribbean | 8 | 8 | 0 |
| Rest Centrally Planned Asia | 9 | 9 | 0 |
| Middle East and North Africa | 10 | 10 | 0 |
| Sub-Saharan Africa | 11 | 12 | 1 |
| South Asia | 12 | 11 | -1 |

To validate the model, the 2022 **E-Government Development Index (EGDI)** values for the assessed countries were collected and aggregated to the regional level using a population-weighted average. These observed values were then compared with the model’s projected digital transformation levels for 2022. As our model projects in 5-year intervals, the 2022 values were obtained via interpolation. Differences between actual and projected values are illustrated in Supplementary Figure 7. The root mean squared error (RMSE) and mean absolute error (MAE) for the comparison are 0.0254 and 0.0206, respectively.

**Supplementary Table 8: Permutation feature and moment-independent importance measure**

| **Variable** | **Permutation Feature Importance** | **Moment-Independent Importance Measure** |
| --- | --- | --- |
| GDP per capita, PPP (2017) | 0.083301  (95 % CI [0.065641, 0.086451]) | 18.591638 |
| Population | 0.010685  (95 % CI [0.003647, 0.017384]) | 0.207159 |
| R&D expenditure (% of GDP) | -0.003672  (95 % CI [-0.005202, 0.000072]) | 0.174024 |

We used two approaches to assess each variable’s global influence on predictions. Permutation feature importance is conducted by randomly shuffling each input variable while keeping others fixed, and then determining the resulting increase in root mean squared error. A higher value increase indicates greater importance, as it shows the model relies. The 95 % confidence interval is reported for better transparency. R&D expenditure (% of GDP) has a confidence interval includes zero, suggesting that its effect may be statistically indistinct or potentially underestimated due to correlation.

To address the potential limitations of permutation importance (random shuffling approach) in the presence of correlated variables, moment-independent sensitivity analysis is conducted. For each input variable, we fixed its value at selected quantiles (25^th^, 50^th^, 75^th^) while leaving all other inputs unchanged. We then estimated the output distributions via kernel density estimation and quantify the divergence from the original predictions using symmetric Kullback–Leibler divergence (comparing shape of new predictions to the original one). The greater the average deviation, the more influential the variable.

Both approaches show that the predictive model is globally most sensitive to GDP per capita, PPP (2017). The sensitivity scores reported in Supplementary Table 8 represent global model-level importance values, based on how each input variable affects the of predicted outcomes (digital transformation level). These values reflect the average model dependence on each input across the entire panel (rather than country or year specific effects).

**Supplementary Table 9:** **Historical model explaining R&D expenditure used to project future R&D and relative digital transformation levels**.

A random effects model was preferred to fixed effects based on results of the Hausman test, implying that the unobserved country-specific factors are not systematically related to our independent variable. Such factors could be innovation culture or institutional framework, which may affect R&D expenditure expressed as % of GDP, independently of economic status. There are countries with relatively high GDP per capita and R&D expenditure, such as Singapore and Israel (high even compared to other high-income countries – possibly due to cultural innovation, institutional framework or geopolitical) or low R&D expenditure like Luxembourg,

GDP per capita as an independent variable of random effect model is tested and applied as the input for future projections, GDP per capita in square term and GDP per capita growth was tested as a sensitivity check and the differences of the estimation using Argentina as an example is illustrated below. Results were similar.

| **Random Effect Model 1 (Tested and Applied)** | | **Random Effect Model 2 (Tested)** | |
| --- | --- | --- | --- |
| *R&D Expenditure_it_* $=\alpha+\gamma_{i}+\beta_{1}{GDP}_{it} +\varepsilon_{it}$  in which $\alpha$represents the overall intercept,$\gamma_{i}$ represents the random effect specific to entity or country $i$, $\varepsilon_{it}$ is the error term for entity $i$ at time $t$  $\beta_{1}$= coefficient for GDP per capita, PPP (2017) = 2.89 ×10^-5^  *p*-value <0.05, R^2^ = 0.6861 | | *R&D Expenditure_it_* $=\alpha+\gamma_{i}+\beta_{1}{GDP}_{it} +\beta_{2}{(GDP}_{it})^{2}+\varepsilon_{it}$  in which $\alpha$represents the overall intercept,$\gamma_{i}$ represents the random effect specific to entity or country $i$, $\varepsilon_{it}$ is the error term for entity $i$ at time $t$  $\beta_{1}$= coefficient for GDP per capita, PPP (2017) = 5.858 ×10^-5^  $\beta_{2}$= coefficient for square of GDP per capita, PPP (2017) = -3.82 ×10^-10^  *p*-value <0.05, R^2^ = 0.8005 | |
| **Both models result in small differences in predicted digital transformation. For example, by region in 2050 (SSP2)** | | | |
| Region | **Digital Transformation Level -Random Effect Model 1** | | **Digital Transformation Level -Random Effect Model 2** |
| Central and Eastern Europe | 1.151170 | | 1.153513 |
| China | 1.001592 | | 1.003908 |
| Former Soviet Union | 0.967486 | | 0.968862 |
| Latin America and the Caribbean | 0.959320 | | 0.960714 |
| Middle East and North Africa | 0.858649 | | 0.860095 |
| North America | 1.271782 | | 1.272611 |
| Other Pacific Asia | 1.112362 | | 1.113798 |
| Pacific OECD | 1.186669 | | 1.188226 |
| Rest Centrally Planned Asia | 0.989109 | | 0.991225 |
| South Asia | 0.963034 | | 0.963490 |
| Sub-Saharan Africa | 0.653647 | | 0.653877 |
| Western Europe | 1.175740 | | 1.177365 |

**Supplementary Table 10: Interpreting projected relative digital transformation levels.**

| Interpreting Relative Digital Transformation Levels |
| --- |
| The E-Government Development Index (EGDI) described in Supplementary Table 4 is an index over the range 0-1. This creates a saturation effect as values approach the upper limit, constraining the potential to measure further digital transformation. Countries or regions like the US or North America are already close to 1 based on their historical performance.  In our projections, we remove the restriction of the index scale being capped at 1. This aligns with our goal of capturing future change in the relative levels of digital transformation between countries or regions and over time. However, caution is needed in the application and interpretation of these projections to avoid misrepresentation. In particular, our projected digital transformation levels should only be used to compare changing performance *relative* to other countries or regions, and over time. Conversely our projected digital transformation levels should not be used as a measure of future EGDI, nor should it be used as a measure of *absolute* performance of any given country.  An alternative approach would have been to denormalise and rescale the historical EGDI. This approach was used by Andrijevic et al. (2020) for their governance index in which they used an adjusted min-max approach, followed by applying a beta model to ensure projected values remained within the 0-1 range. However, this approach requires having the underlying information on all the indices and sub-indicators in their original units. This is not available in our case. |

**Supplementary Table 11: Categorisation of digital transformation levels based on 2020-2050 data from 62 countries.**

| **Category** | **Percentile** | **Digital Transformation Percentile Thresholds** |
| --- | --- | --- |
| very high | > 90th | >1.2194 |
| high | > 75 and ≤ 90^th^ | >1.0950 and ≤1.2194 |
| medium | > 50 and ≤75th | >0.9560 and ≤1.0950 |
| low | >25 and ≤ 50th | >0.8344 and ≤ 0.9560 |
| very low | ≤ 25th | ≤0.3095 |

**Supplementary Table 12: Digital transformation level percentiles by number of countries and population (62 Countries dataset).**

| **Year** | **Scenario** | **Percentile** | **Number of Countries** | **Population** | **Percentage by number of countries (%)** | **Percentage by Population (%)** |
| --- | --- | --- | --- | --- | --- | --- |
| 2050 | SSP1 | Very High | 23 | 9.48 × 10⁸ |  |  |
| 2050 | SSP1 | High | 17 | 3.07 × 10⁸ |  |  |
| 2050 | SSP1 | Medium | 13 | 2.31 × 10⁹ |  |  |
| 2050 | SSP1 | Low | 6 | 1.68 × 10⁹ | 14.52 | 34.06 |
| 2050 | SSP1 | Very Low | 3 | 1.58 × 10⁸ |  |  |
| 2050 | SSP2 | Very High | 17 | 6.37 × 10⁸ |  |  |
| 2050 | SSP2 | High | 18 | 5.17 × 10⁸ |  |  |
| 2050 | SSP2 | Medium | 14 | 3.63 × 10⁹ |  |  |
| 2050 | SSP2 | Low | 8 | 4.30 × 10⁸ | 20.9 | 12.92 |
| 2050 | SSP2 | Very Low | 5 | 2.81 × 10⁸ |  |  |
| 2050 | SSP3 | Very High | 4 | 1.28 × 10⁷ |  |  |
| 2050 | SSP3 | High | 18 | 8.19 × 10⁸ |  |  |
| 2050 | SSP3 | Medium | 22 | 2.27 × 10⁹ |  |  |
| 2050 | SSP3 | Low | 9 | 2.03 × 10⁹ | 29.03 | 45.07 |
| 2050 | SSP3 | Very Low | 9 | 5.17 × 10⁸ |  |  |
| 2050 | SSP4 | Very High | 22 | 8.03 × 10⁸ |  |  |
| 2050 | SSP4 | High | 17 | 3.73 × 10⁸ |  |  |
| 2050 | SSP4 | Medium | 11 | 3.57 × 10⁹ |  |  |
| 2050 | SSP4 | Low | 6 | 3.24 × 10⁸ | 19.35 | 14.20 |
| 2050 | SSP4 | Very Low | 6 | 4.61 × 10⁸ |  |  |
| 2050 | SSP5 | Very High | 36 | 1.23 × 10⁹ |  |  |
| 2050 | SSP5 | High | 9 | 1.67 × 10⁹ |  |  |
| 2050 | SSP5 | Medium | 11 | 2.28 × 10⁹ |  |  |
| 2050 | SSP5 | Low | 4 | 1.36 × 10⁸ | 9.68 | 15.18 |
| 2050 | SSP5 | Very Low | 2 | 1.46 × 10⁸ |  |  |

**Supplementary Fig. 1: Digital transformation levels in 12 world regions (population weighted) within five shared socioeconomic pathways (SSPs) up to 2100.**

| 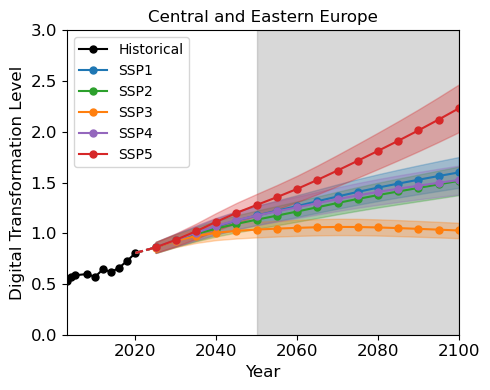**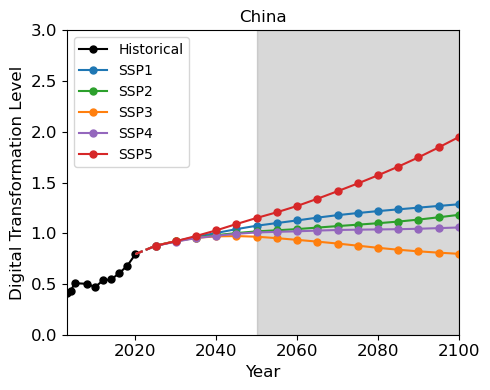 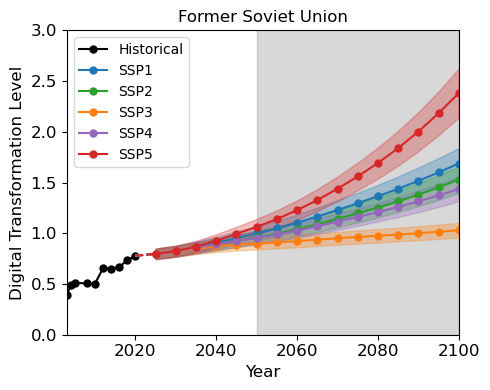** |
| --- |
| **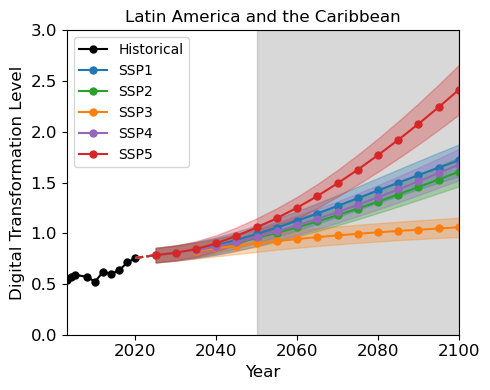 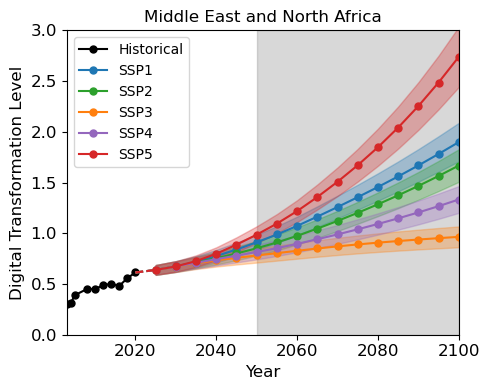 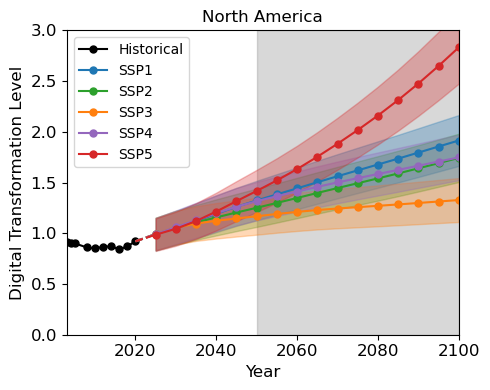**  **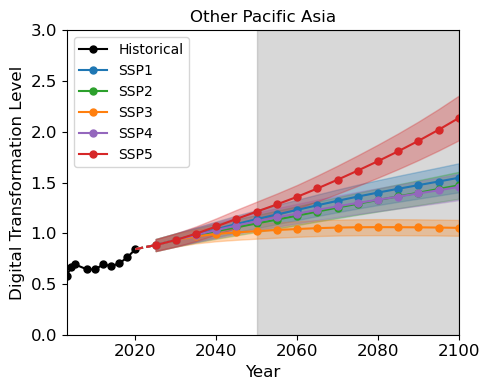 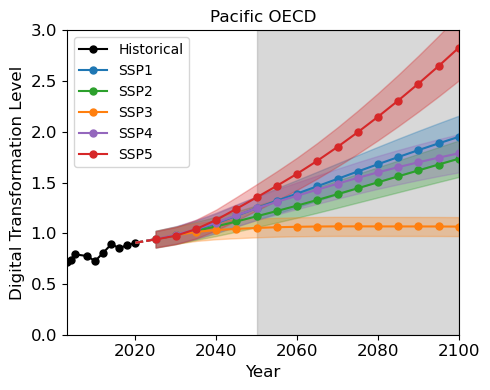 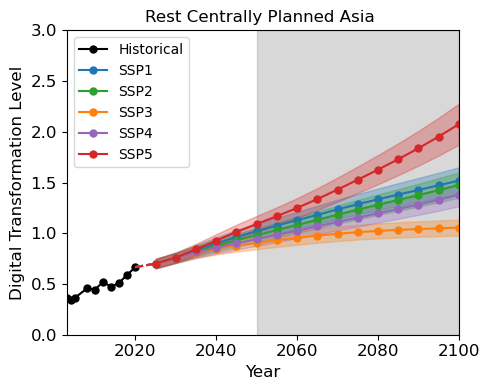** |
| **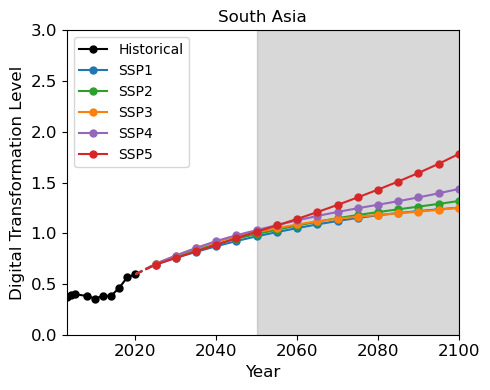 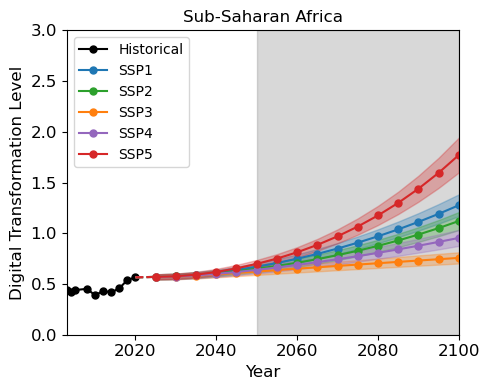 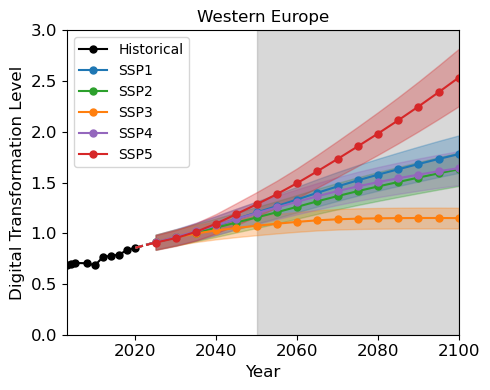** |

**Supplementary Fig. 2: Digital transformation levels (2050) categorised by percentiles across 5 SSPs and by GDP/cap based on 180 countries dataset (a)** Percentile categories are defined based on the data distribution of all 180 countries, capturing each country's relative level of digital transformation within the full dataset. This approach is better suited for interpreting relative performance across the entire group of countries. **(b)** Percentile categories are fixed using thresholds derived from the data distribution of 62 countries, as defined in Supplementary 9. This method is better for direct comparisons among the 62 countries, as the category cutoffs remain consistent. However, it does not reflect the relative standing of countries within the broader group of 180.

(a) Percentile categories are defined based on the data distribution of all 180 countries


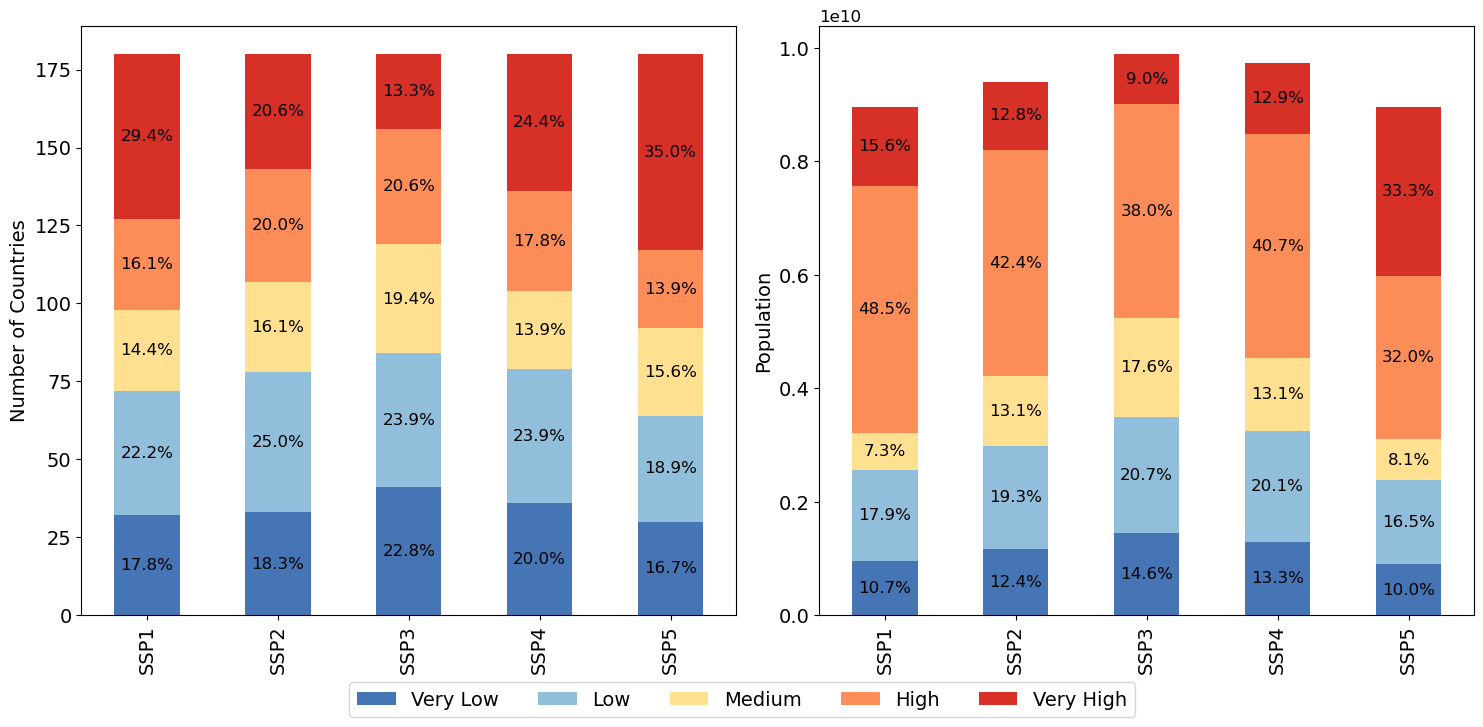


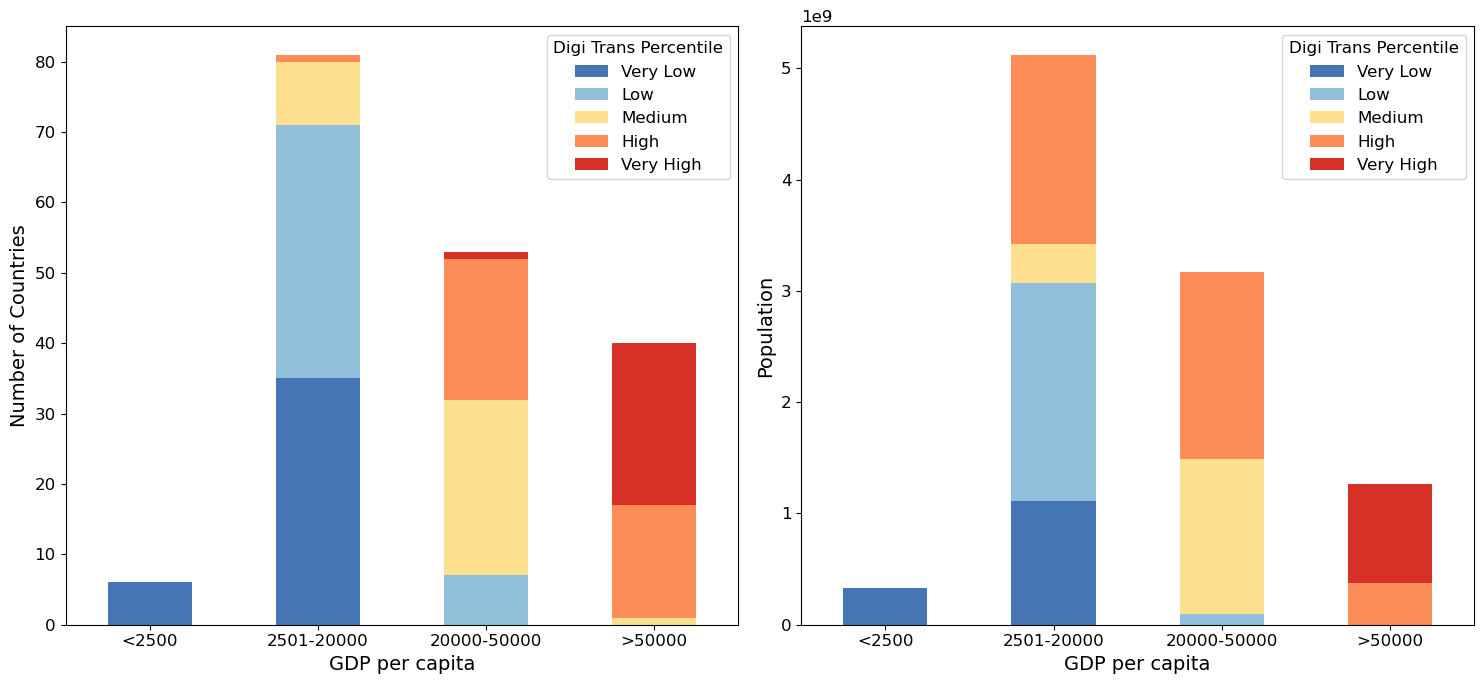


(b) Percentile categories are fixed using thresholds derived from the data distribution of 62 countries, as defined in Supplementary Table 11

**
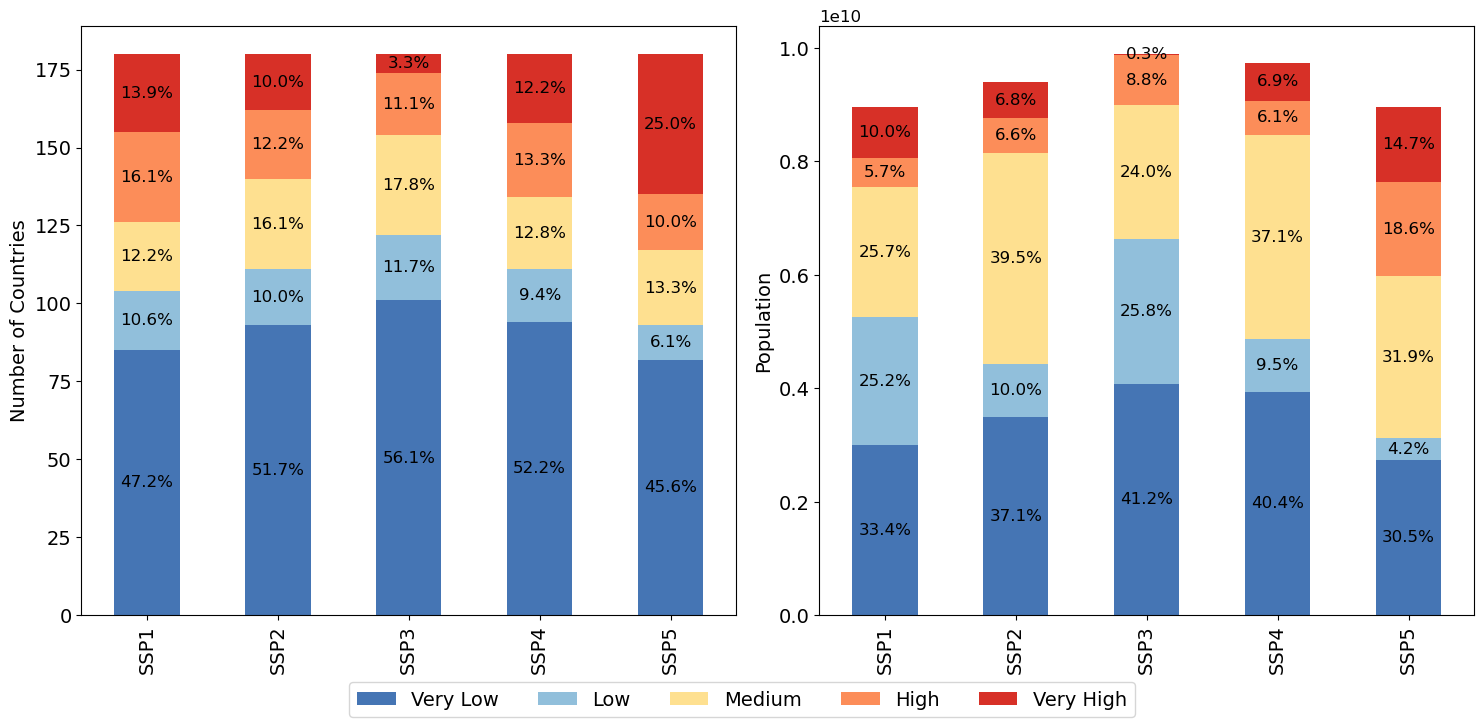
**

**
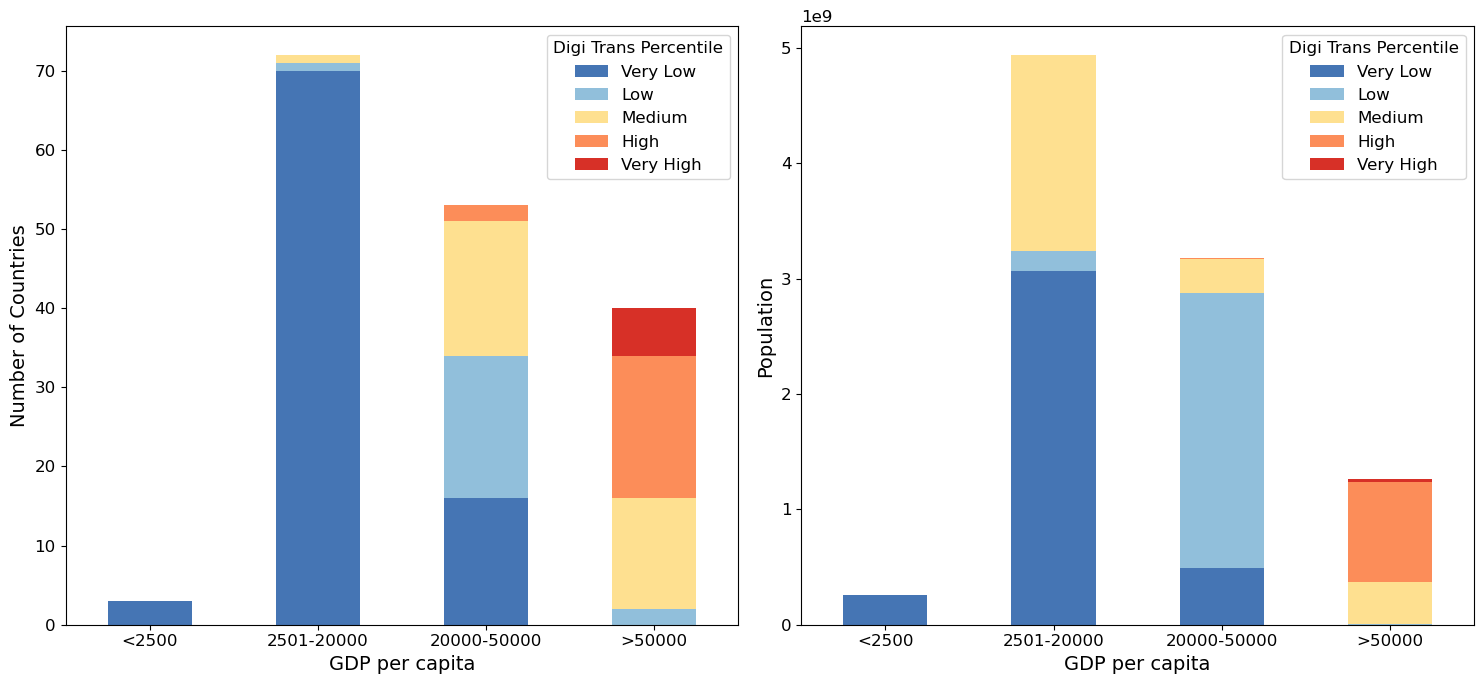
**

**Supplementary Fig. 3: Workflow for projecting digital transformation levels under SSP-consistent assumptions.**


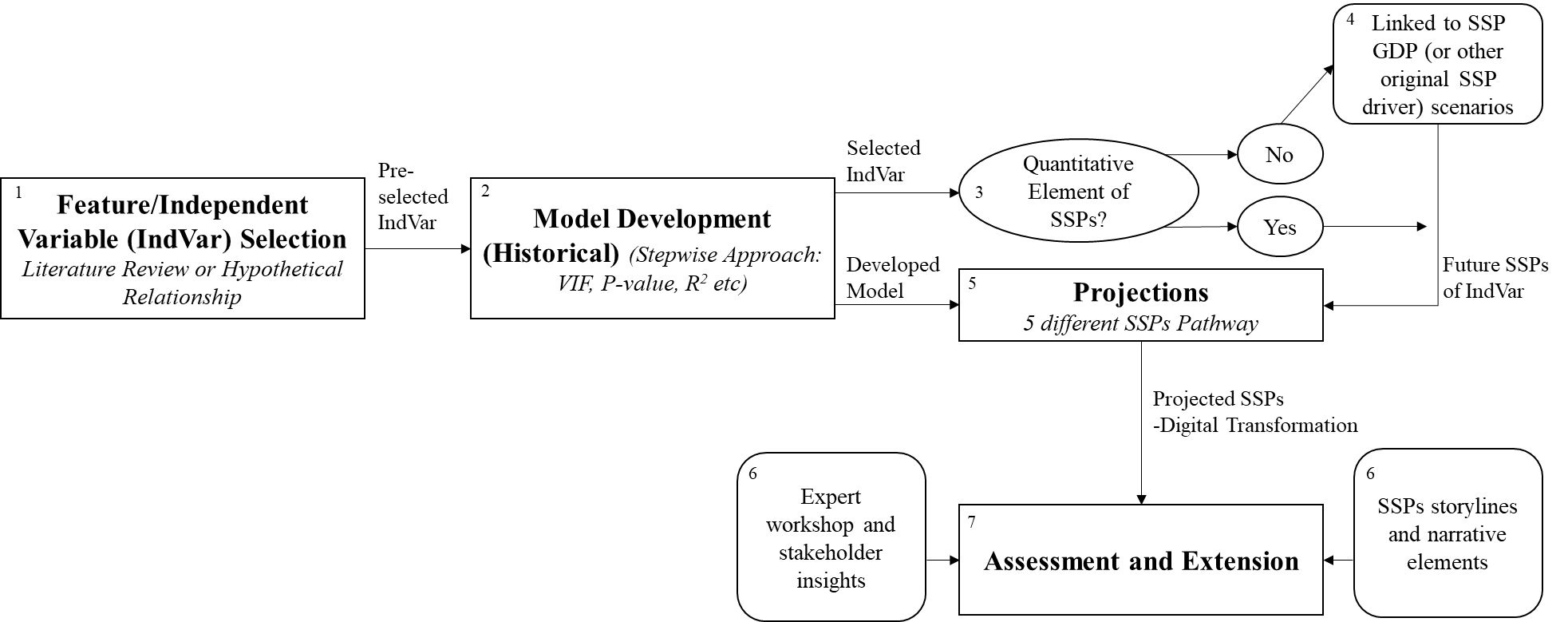


**Supplementary Fig. 4: Assessed countries and world regions.**


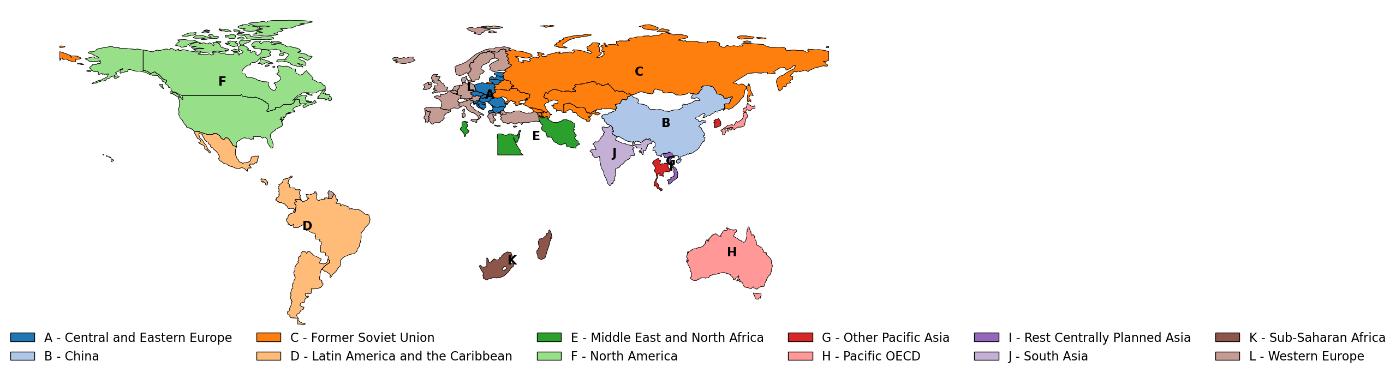


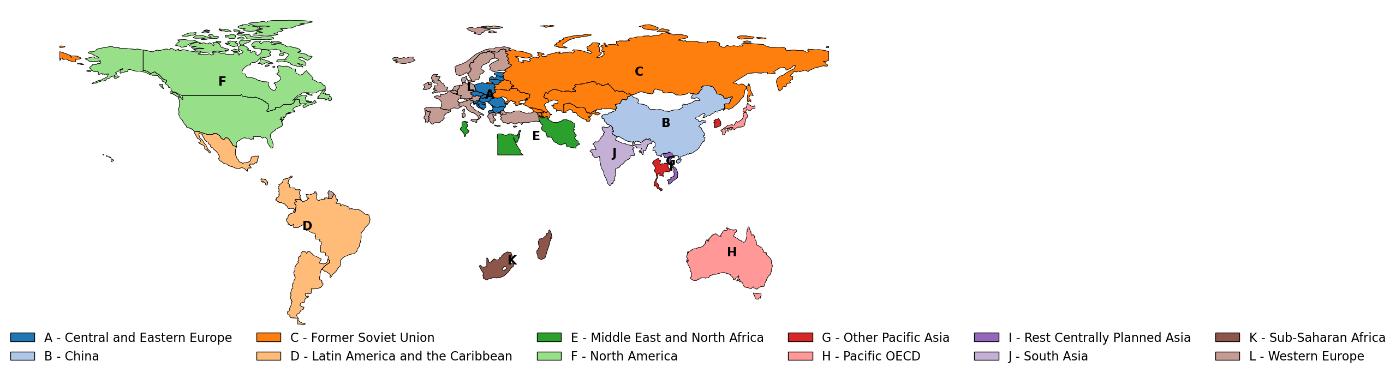


| **Region** | **Countries** | **2020** | | |
| --- | --- | --- | --- | --- |
|  |  | **GDP** | **Average GDP/Cap** | **Population** |
| Central and Eastern Europe (13) | 'Bosnia and Herz.', 'Bulgaria', 'Croatia', 'Czechia', 'Estonia', 'Hungary', 'Latvia', 'Lithuania', 'North Macedonia', 'Poland', 'Romania', 'Slovakia', 'Slovenia' | 3.31e+12 | 30792 | 107571062 |
| China (1) | 'China' | 2.30e+13 | 16297 | 1411100000 |
| Former Soviet Union (8) | 'Armenia', 'Azerbaijan', 'Belarus', 'Georgia', 'Kazakhstan', 'Russia Federation', 'Ukraine', 'Uzbekistan' | 5.52e+12 | 20643 | 267194301 |
| Latin America and the Caribbean (5) | 'Argentina', 'Brazil', 'Colombia', 'Costa Rica', 'Mexico' | 7.03e+12 | 15959 | 440625136 |
| Middle East and North Africa (4) | 'Egypt', 'Iran', 'Israel', 'Tunisia' | 3.04e+12 | 14047 | 216132150 |
| North America (2) | 'Canada', 'United States of America' | 2.17e+13 | 58722 | 369518678 |
| Other Pacific Asia (3) | 'Singapore', 'South Korea', 'Thailand' | 3.94e+12 | 30565 | 128997710 |
| Pacific OECD (2) | 'Australia', 'Japan' | 6.30e+12 | 41498 | 151910248 |
| Rest Centrally Planned Asia (1) | 'Vietnam' | 1.01e+12 | 10451 | 96648685 |
| South Asia (1) | 'India' | 8.62e+12 | 6172 | 1396387127 |
| Sub-Saharan Africa (2) | 'Madagascar', 'South Africa' | 7.97e+11 | 9159 | 87027104 |
| Western Europe (20) | 'Austria', 'Belgium', 'Cyprus', 'Denmark', 'Finland', 'France', 'Germany', 'Greece', 'Iceland', 'Ireland', 'Italy', 'Luxembourg', 'Malta', 'Netherlands', 'Norway', 'Portugal', 'Spain', 'Sweden', 'Turkey', 'United Kingdom' | 1.99e+13 | 39878 | 498828691 |

The selection of countries for analysis relies on listwise deletion, whereby any observation containing one or more missing values is omitted entirely. The timeframe considered is 2003-2020. A total of 62 countries are included, representing 12 model regions defined by the MESSAGEix GLOBIOM model: Central and Eastern Europe, China, Former Soviet Union, Latin America and the Caribbean, Middle East and North Africa, North America, Other Pacific Asia, Pacific OECD, Rest Centrally Planned Asia, South Asia, Sub-Saharan Africa, and Western Europe.

**Supplementary Fig. 5: Projected future R&D expenditure in 12 world regions (GDP-weighted country aggregations) within five shared socioeconomic pathways (SSPs).**

| 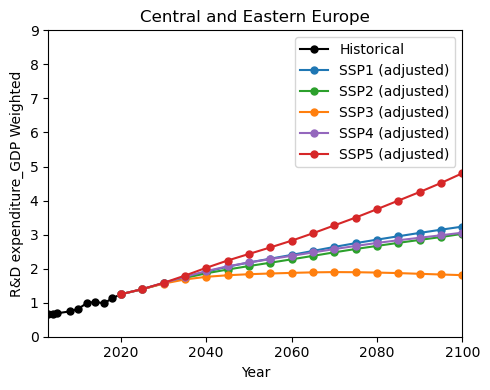 | 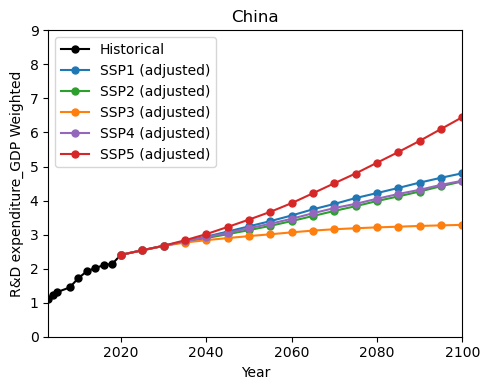 | 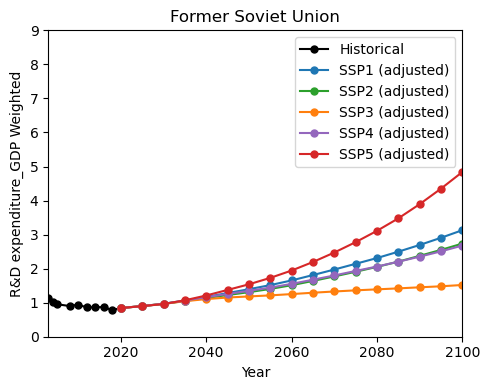 |
| --- | --- | --- |
| 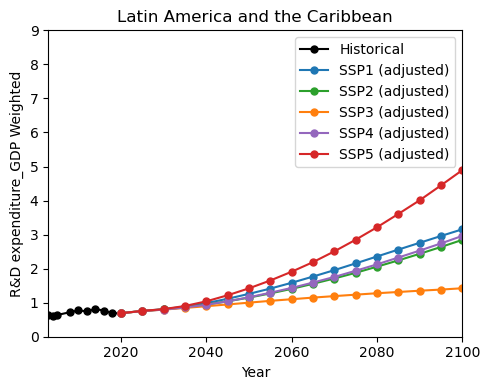 | 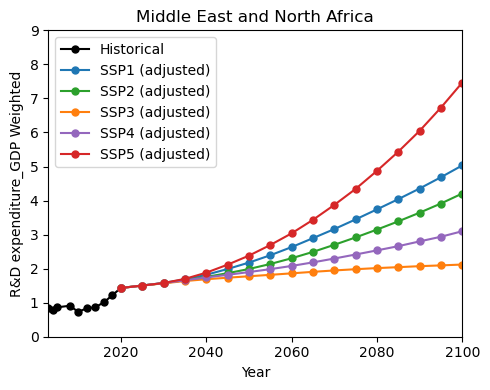 | 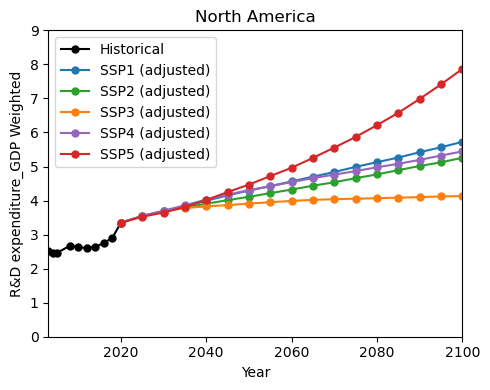 |
| 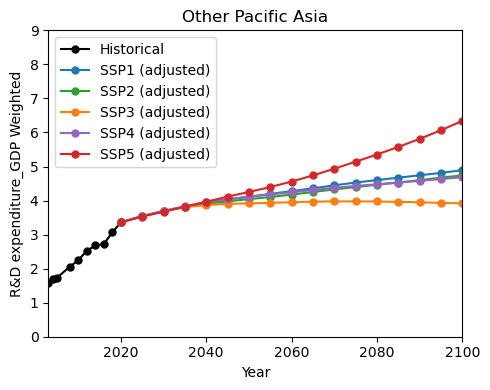 | 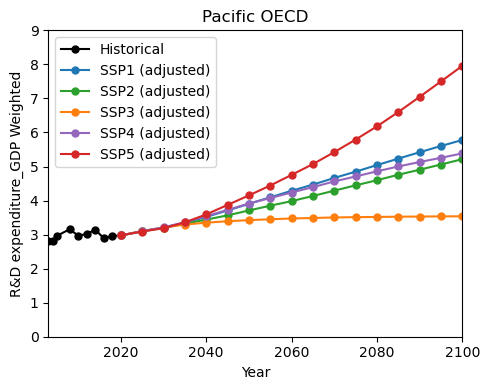 | 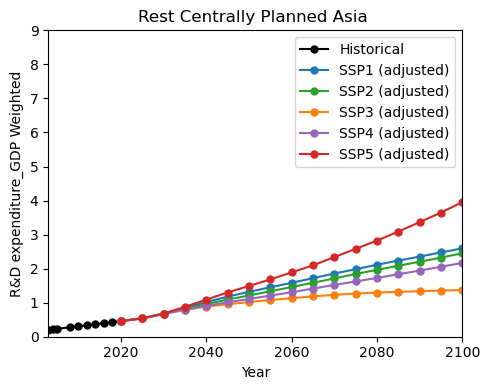 |
| 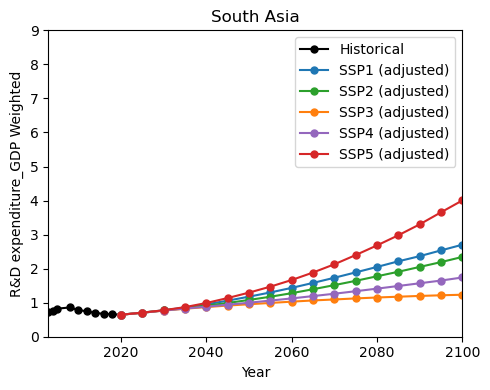 | 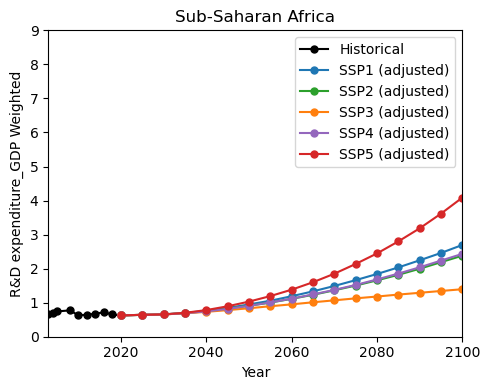 | 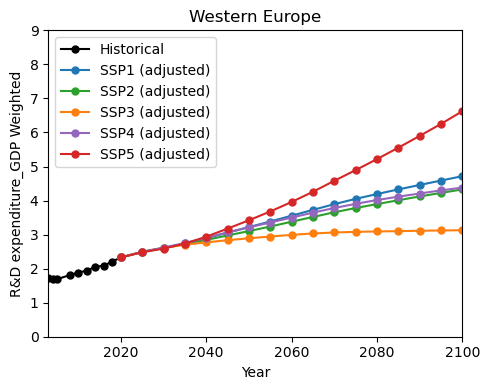 |

**Supplementary Fig. 6: Aligning historical trends and future projections using 2020 as a calibration point.** Data for the Western Europe region is shown as an example.
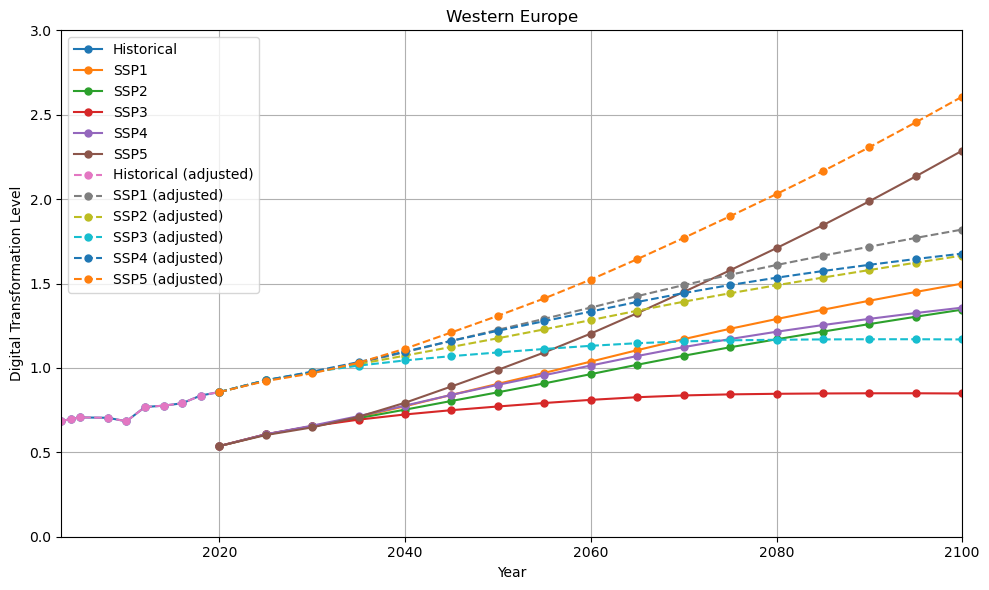


A baseline adjustment was applied using the year 2020 as the reference point. The difference between the model’s projected value for 2020 and the actual observed value was calculated, and this difference was added uniformly to all projected values from 2050 to 2100. This adjustment preserves the model’s estimated trajectory (relative trends) while aligning the projection with the historical data at the transition point.

**Supplementary Fig 7: Comparison of actual and projected digital transformation level in 2022.** Refer to Supplementary Table 7 for RSME and MAE.


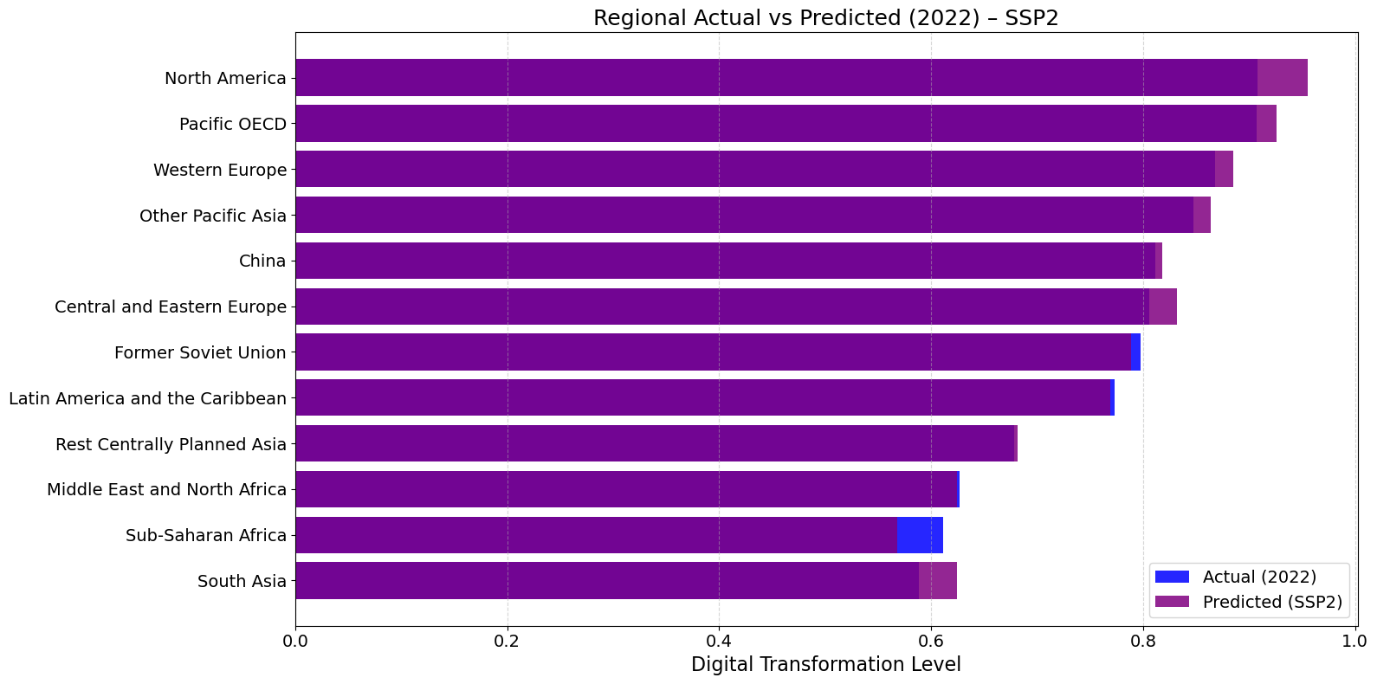


**Supplementary Information: Out-of-Sample or Generalised Projection**

The model for regional projection is trained based on 62 countries to represent the 12 regions. This approach is followed given historical data constraints (data availability, specifically the R&D expenditure is not available) for the other countries. To provide a wider view on what it means in term of number of population and country reside in different digital transformation level globally, we use the same model to project the potential future for 180 countries (additional of 118 countries) and discuss side by side with the results for 62 countries for perspective giving (see section: “Uneven digital transformation across world regions”). We report projected changes in relative digital transformation levels in all 180 countries in a supplementary Excel file. For this, we assume that the 118 added countries exhibit similar relationships (GDP per capita, population, R&D expenditure) to those of their respective regions, recognising that this approach will not capture country specificities.

As this involved generalisation and out-of-sample projection, we assessed whether the model is sufficiently generalisable to estimate digital transformation levels for the additional countries. Cumulative distribution functions plots are constructed to illustrate the distribution and inform about potential biases and extrapolation risks, where a similar distribution indicates lower introduced bias and uncertainty in projection. Table A show the list of countries that have increased from the original dataset, which was represented by 62 countries as listed in Supplementary Figure 4. Using 70 % as the cut-off threshold, the regions that may not be well represented for generalisation are Middle East and North Africa, Other Pacific Asia and Sub-Saharan Africa (Table B).

**Supplementary Table 13:** Countries and socioeconomic indicators included in the extended datasets, with 2020 data used as historical data for model development as an example

| **Region and Countries** | **2020** | | |
| --- | --- | --- | --- |
|  | **GDP** | **Average GDP/cap** | **Population** |
| Central and Eastern Europe (16):  'Albania', 'Bosnia and Herzegovina', 'Bulgaria', 'Croatia', 'Czechia', 'Estonia', 'Hungary', 'Latvia', 'Lithuania', 'Montenegro', 'North Macedonia', 'Poland', 'Romania', 'Serbia', 'Slovakia', 'Slovenia' | 3.35e+12 | 28071 | 1.19e+08 |
| China (1): 'China' | 2.30e+13 | 16297 | 1.41e+09 |
| Former Soviet Union (12):  'Armenia', 'Azerbaijan', 'Belarus', 'Georgia', 'Kazakhstan', 'Kyrgyzstan', 'Moldova', 'Russian Federation', 'Tajikistan', 'Turkmenistan', 'Ukraine', 'Uzbekistan' | 5.52e+12 | 18804 | 2.94e+08 |
| Latin America and the Caribbean (30):  'Antigua and Barbuda', 'Argentina', 'Bahamas', 'Barbados', 'Belize', 'Bolivia', 'Brazil', 'Chile', 'Colombia', 'Costa Rica', 'Cuba', 'Dominican Republic', 'Ecuador', 'El Salvador', 'Grenada', 'Guatemala', 'Guyana', 'Haiti', 'Honduras', 'Jamaica', 'Mexico', 'Nicaragua', 'Panama', 'Paraguay', 'Peru', 'Saint Lucia', 'Saint Vincent and the Grenadines', 'Suriname', 'Trinidad and Tobago', 'Uruguay' | 8.66e+12 | 14057 | 6.16e+08 |
| Middle East and North Africa (19):  'Algeria', 'Bahrain', 'Egypt', 'Iran', 'Iraq', 'Israel', 'Jordan', 'Kuwait', 'Lebanon', 'Libya', 'Morocco', 'Oman', 'Qatar', 'Saudi Arabia', 'South Sudan', 'Sudan', 'Tunisia', 'United Arab Emirates', 'Yemen' | 7.56e+12 | 15000 | 5.04e+08 |
| North America (2):  'Canada', 'United States of America' | 2.17e+13 | 58722 | 3.70e+08 |
| Other Pacific Asia (17):  'Brunei Darussalam', 'Fiji', 'Indonesia', 'Kiribati', 'Malaysia', 'Micronesia', 'Myanmar', 'Papua New Guinea', 'Philippines', 'Samoa', 'Singapore', 'Solomon Islands', 'South Korea', 'Thailand', 'Timor-Leste', 'Tonga', 'Vanuatu' | 9.12e+12 | 14910 | 6.12e+08 |
| Pacific OECD (3):  'Australia', 'Japan', 'New Zealand' | 6.51e+12 | 41713 | 1.56e+08 |
| Rest Centrally Planned Asia (5):  'Cambodia', 'Laos', 'Mongolia', 'North Korea', 'Viet Nam' | 1.22e+12 | 8208 | 1.49e+08 |
| South Asia (7):  'Bangladesh', 'Bhutan', 'India', 'Maldives', 'Nepal', 'Pakistan', 'Sri Lanka' | 1.11e+13 | 6032 | 1.83e+09 |
| Sub-Saharan Africa (47):  'Angola', 'Benin', 'Botswana', 'Burkina Faso', 'Burundi', 'Cabo Verde', 'Cameroon', 'Central African Republic', 'Chad', 'Comoros', 'Congo', "Cote d'Ivoire", 'Democratic Republic of the Congo', 'Djibouti', 'Equatorial Guinea', 'Eritrea', 'Eswatini', 'Ethiopia', 'Gabon', 'Gambia', 'Ghana', 'Guinea', 'Guinea-Bissau', 'Kenya', 'Lesotho', 'Liberia', 'Madagascar', 'Malawi', 'Mali', 'Mauritania', 'Mauritius', 'Mozambique', 'Namibia', 'Niger', 'Nigeria', 'Rwanda', 'Sao Tome and Principe', 'Senegal', 'Seychelles', 'Sierra Leone', 'Somalia', 'South Africa', 'Tanzania', 'Togo', 'Uganda', 'Zambia', 'Zimbabwe' | 4.03e+12 | 3718 | 1.08e+09 |
| Western Europe (21):  'Austria', 'Belgium', 'Cyprus', 'Denmark', 'Finland', 'France', 'Germany', 'Greece', 'Iceland', 'Ireland', 'Italy', 'Luxembourg', 'Malta', 'Netherlands', 'Norway', 'Portugal', 'Spain', 'Sweden', 'Switzerland', 'Turkey', 'United Kingdom' | 2.05e+13 | 40323 | 5.07e+08 |

**Supplementary Table 14:** The percentage share of the number of countries, GDP, and population of the 62-country dataset compared to the 180-country dataset, categorised by region.

| **Regions** | **Number of represented countries in 62 countries dataset** | **Number of countries in the 180 countries dataset** | **% of the total 180 countries** | **% of the GDP of 180 countries** | **% of the Population of 180 countries** |
| --- | --- | --- | --- | --- | --- |
| Central and Eastern Europe | 13 | 16 | 81.25 | 99 | 90 |
| China | 1 | 1 | 100 | 100 | 100 |
| Former Soviet Union | 8 | 12 | 66.67 | 99 | 91 |
| Latin America and the Caribbean | 5 | 30 | 16.67 | 81 | 72 |
| Middle East and North Africa | 4 | 19 | 21.05 | 40 | 43 |
| North America | 2 | 2 | 100 | 100 | 100 |
| Other Pacific Asia | 3 | 17 | 17.65 | 43 | 21 |
| Pacific OECD | 2 | 3 | 66.67 | 96 | 97 |
| Rest Centrally Planned Asia | 1 | 5 | 20 | 83 | 65 |
| South Asia | 1 | 7 | 14.29 | 78 | 76 |
| Sub-Saharan Africa | 2 | 47 | 4.26 | 20 | 8 |
| Western Europe | 20 | 21 | 95.23 | 97 | 98 |

Figure A shows the overall distribution of GDP and population in 2020 for the 62 countries used for model development and the data distribution of the 180-country dataset, where 118 countries are out of sample. In general, despite covering a similar range of minimum and maximum values, the 62-country dataset is biased toward wealthier countries with higher GDP per capita, making it less representative of the global or regional context. For example, 80% of the 62-country dataset has a GDP per capita less than or equal to 48,652, compared to the 180-country dataset, where 80% of countries have a GDP per capita less than or equal to 32,311. This bias suggests that the model will likely overpredict digital transformation levels for regions with lower GDP per capita, which are underrepresented in the 62-country dataset. Therefore, caution is needed when interpreting the projected digital transformation levels for out-of-sample countries. As the model is biased towards wealthier countries, the actual number of countries and populations with low digital transformation could be even higher than reported. The model is likely to generalise well when predicting population-related effects on digital transformation for out-of-sample countries, as the population distribution between the two datasets is relatively similar.


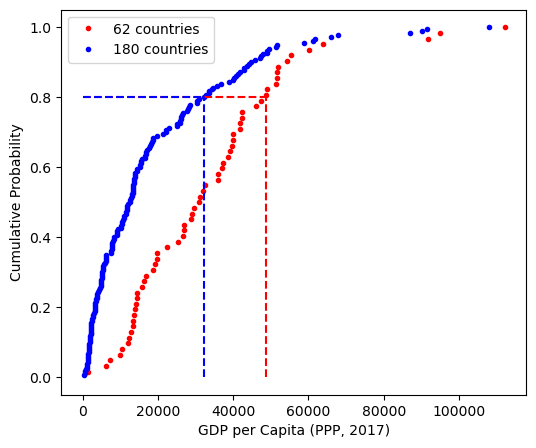

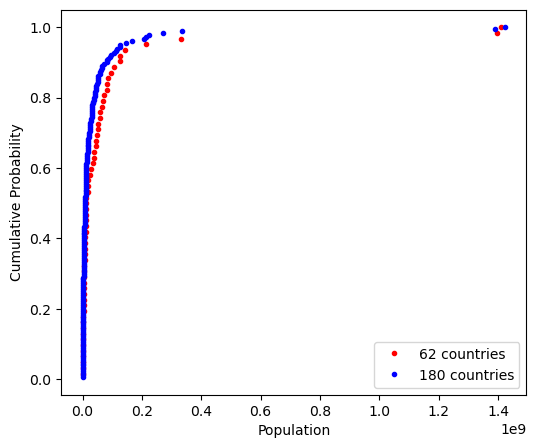


**Supplementary Figure 8:** Cumulative Distribution Function (CDF) plots of GDP per capita and population for the 62-country dataset (red) and the 180-country dataset (blue) in 2020
